# Perceptions of the use of biomarkers for Alzheimer's disease diagnosis: A systematic review and synthesis of the qualitative literature

**DOI:** 10.1002/dad2.70380

**Published:** 2026-06-03

**Authors:** Jemma Hazan, Mitchell Mealing, Emma Mittelman, Sarah Godsell, Sophie Roche, Fabiana Lorencatto, Penny Rapaport, Ashvini Keshavan, Jonathan M. Schott, Robert Howard

**Affiliations:** ^1^ Division of Psychiatry University College London London UK; ^2^ Royal Free London NHS Foundation Trust London UK; ^3^ North London NHS Foundation Trust London UK; ^4^ Centre for Behaviour Change, Department of Clinical, Educational and Health Psychology University College London London UK; ^5^ Dementia Research Centre, UCL Queen Square Institute of Neurology University College London London UK; ^6^ UK Dementia Research Institute University College London London UK

**Keywords:** Alzheimer's disease, biomarker, dementia, diagnosis, qualitative research, review, theoretical domains framework

## Abstract

Biomarker investigations are increasingly used in the diagnostic assessment of Alzheimer's disease (AD), yet stakeholder perceptions and implementation factors remain underexplored. No systematic qualitative synthesis exists on these views. This systematic review aimed to synthesize the qualitative literature on perceptions of diagnostic AD biomarker investigations in both clinical and research settings among individuals with cognitive impairment, caregivers, and health‐care professionals (HCPs) and to identify barriers and enablers to their practical implementation. A systematic review searched four databases up to May 2025 for English‐language qualitative studies on stakeholder (HCPs, caregivers, patients) perspectives. A meta‐ethnographic approach synthesized findings, with barriers/enablers classified using the theoretical domains framework (TDF). From 4319 records, 26 studies were included, yielding five key concepts: stakeholder expectations, test result significance, shared decision‐making, diagnostic certainty, and test delivery systems. Barriers included lack of understanding (knowledge) and emotional burden (emotion). Enablers involved supporting decision making (memory, attention, decision processes) and beliefs about testing benefits (beliefs about consequences), though risks were equally noted. Barriers to AD biomarker use involve multiple TDF domains, with contrasting stakeholder viewpoints. Improving knowledge of biomarkers and addressing perceived benefits and risks can guide interventions, promoting more effective test use.

## Highlights

- This is the first known systematic qualitative review to synthesize stakeholder perceptions on Alzheimer's disease (AD) biomarker testing, using a meta‐ethnographic approach.
- We describe an “uncertainty feedback loop”: Testing, intended to manage uncertainty, may paradoxically introduce new uncertainties, highlighting challenges in interpreting tests and communicating results.
- Barriers and enablers to the use of AD biomarker investigation were classified using the theoretical domains framework, with varied stakeholder perspectives on testing. Barriers included lack of understanding (knowledge) and emotional burden (emotion). Enablers involved supporting decision making (memory, attention, decision processes) and beliefs about testing benefits (beliefs about consequences), though stakeholders also noted potential risks.
- Interventions and policies should address these barriers and leverage enablers, including evidence‐based training programs, guidelines, and communication frameworks to structure testing discussions.

## 1 BACKGROUND

Delays in diagnosing Alzheimer's disease (AD) and high rates of misdiagnosis remain significant challenges within health‐care systems.^1^ The average time period between symptom onset and diagnosis can be > 3.5 years.^2^ This can delay access to evidence‐based treatments and post‐diagnostic support for patients and caregivers. AD biomarker investigations, such as cerebrospinal fluid (CSF) analysis and neuroimaging techniques including amyloid and tau positron emission tomography (PET), are currently used infrequently in the diagnostic assessment of individuals with cognitive difficulties.^3^ In the UK, < 2% of patients underwent amyloid PET or CSF analysis in 2023.^3^ These methods can detect the in vivo neuropathological hallmarks of AD—amyloid deposition and tau pathology—with the potential to enable a more definitive diagnosis and rule out AD as a contributing factor to a patient's symptoms.^4^ These methods also are prerequisites for emerging disease‐modifying therapy use.^5^ To date, the most cited barriers to their use have been resource limitations, test invasiveness, and insufficient training in test administration.^6^

Recent advancements in blood biomarker detection could significantly expand access to biomarker investigations by enabling easier, cheaper, and less invasive testing. However, in the UK, integrating these technologies into National Health Service (NHS) memory services, in which biomarker investigations have been rarely used, presents challenges. These include gaps in knowledge, required changes to infrastructure, and the need for staff training to support their implementation.^7^ Additionally, there are ethical and social considerations including the prognostic value of testing in mild cognitive impairment (MCI),^8^ the psychological impact of disclosure,^9^ and equitable access to testing.^3^ Characterizing these challenges and understanding the perceptions of patients, caregivers, and health‐care professionals (HCPs) is essential to support successful implementation of these innovations. Qualitative research can provide valuable insights into the complexities of health‐care delivery. While several qualitative studies have explored stakeholder views on AD biomarker use,^7^, ^10^ a comprehensive synthesis of these findings has yet to be conducted. The most commonly applied approach for this is meta‐ethnography, which can provide key theoretical and conceptual contributions to advance health‐care policy and practice.^11^ Meta‐ethnography is an inductive, interpretative approach that forms the basis for most qualitative synthesis methods, and is widely used in health‐care research.^12^

Implementing changes in clinical practice (e.g., adoption of a new biomarker test) will require behavioral shifts among stakeholders.^13^ This includes changes in HCP behavior (e.g., choosing when to use the test), in addition to changes in patients and caregivers (e.g., deciding if they want to have a test). Such changes can be conceptualized through the application of behavior change theories and frameworks. Implementation of AD blood‐based biomarker testing will involve multiple interacting behaviors across stakeholder groups. Interventions to implement practice changes are more successful when these guide their design.^14^ One integrated behavior change framework widely used to investigate implementation challenges is the theoretical domains framework (TDF).^15^ The TDF synthesizes multiple behavior change theories into 14 theoretical domains, representing the individual, sociocultural, and environmental influences on behavior, such as: knowledge, skills, environmental context and resources, and social professional role and identity. It offers a theory‐driven approach to identifying these factors and has been used as a synthesis framework in systematic reviews across other health‐care contexts.^16^ Table 1 presents an edited summary of the TDF domains and their definitions, adapted from Atkins et al.^15^

**TABLE 1:** Theoretical domains framework (TDF): domains and definitions (adapted from Atkins et al.^15^).

Domain	Definition
Knowledge	An awareness of the existence of something
Skills	An ability or proficiency acquired through practice
Social/professional role and identity	A coherent set of behaviors and displayed personal qualities of an individual in a social or work setting
Beliefs about capabilities	Acceptance of the truth, reality, or validity about an ability, talent, or facility that a person can put to constructive use
Optimism	The confidence that things will happen for the best or that desired goals will be attained
Beliefs about consequences	Acceptance of the truth, reality, or validity about outcomes of a behavior in a given situation
Reinforcement	Increasing the probability of a response by arranging a dependent relationship, or contingency, between the response and a given stimulus
Intentions	A conscious decision to perform a behavior or a resolve to act in a certain way
Goals	Mental representations of outcomes or end states that an individual wants to achieve
Memory, attention, and decision processes	The ability to retain information, focus selectively on aspects of the environment, and choose between two or more alternatives
Environmental context and resources	Any circumstance of a person's situation or environment that discourages or encourages the development of skills and abilities, independence, social competence, and adaptive behavior
Social influences	Those interpersonal processes that can cause individuals to change their thoughts, feelings, or behaviors
Emotion	A complex reaction pattern, involving experiential, behavioral, and physiological elements, by which the individual attempts to deal with a personally significant matter or event
Behavioral regulation	Anything aimed at managing or changing objectively observed or measured actions

The aim of this systematic review is to synthesize findings from the qualitative literature on perceptions of AD biomarker investigations, using meta‐ethnography to inductively develop new interpretations of current behaviors. We focus on diagnostic biomarkers used in both routine clinical care and research settings, in the context of cognitive impairment. These will be used to identify the barriers and enablers to AD biomarker use among patients, caregivers, and HCPs, by mapping these to TDF domains.

## 2 METHODS

The review was conducted in line with Preferred Reporting Items for Systematic Reviews and Meta‐Analyses (PRISMA) guidelines.^17^ A diagrammatic overview of our methods is provided in Figure S1 in supporting information.

### 2.1 Search strategy

We conducted a systematic search of the primary qualitative study literature exploring perceptions of AD biomarker investigations. Four electronic databases (MEDLINE, PsychINFO, EMCARE, and EMBASE) were searched up to May 2024 with search terms related to AD, biomarker investigations, and perceptions. A medical librarian assisted with specific search strategies for each database. An example search strategy for MEDLINE is included in the supporting information. The search strategy combined four key domains: (1) dementia/AD and cognitive impairment; (2) biomarker investigations (blood, plasma, CSF, amyloid PET, and imaging); (3) qualitative study designs; and (4) stakeholder perspectives, including perceptions, experiences, and decision making. An updated subsequent search in May 2025 did not identify any new studies for inclusion.

### 2.2 Eligibility criteria

The inclusion criteria consisted of peer‐reviewed primary studies, published in full and in English. Eligible studies used qualitative methods for both data collection (e.g., interviews, focus groups, or questionnaires) and analysis. Mixed‐methods studies were included only if they incorporated qualitative methods as part of their approach. Questionnaire‐based studies were included solely if the written responses were analyzed using qualitative methodologies. The studies had to report perceptions on AD biomarker tests, for instance viewpoints on consequences or acceptability of testing. These could be from the perspective of any stakeholder (e.g., HCPs, patients, or caregivers). There were no restrictions on country, publication range, or date. We excluded studies which only included patient participants without recorded cognitive impairment. This criterion was chosen to ensure that findings from both clinical and research settings would be most relevant and applicable to real‐world clinical practice. Biomarker testing in asymptomatic individuals involves different ethical and psychosocial considerations that fall outside the scope of this review.^18^ Gray literature was excluded.

### 2.3 Study selection and quality assessment

**FIGURE 1:**
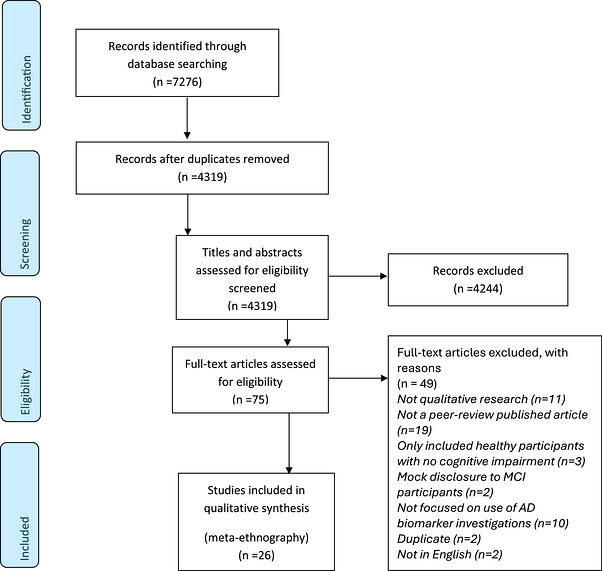
Preferred Reporting Items for Systematic Reviews and Meta‐Analyses flow diagram of search strategy results. AD, Alzheimer's disease; MCI, mild cognitive impairment.

Two reviewers independently screened all titles and abstracts against the eligibility criteria. Full‐text studies were then screened independently by two reviewers, using the same criteria. Any disagreement was resolved at either stage by a review group discussion. An overview of study selection is presented in Figure 1.

The Critical Appraisals Skills Program (CASP) qualitative checklist was used by two reviewers independently to assess study quality.^19^ Further details on the assessment of each domain are provided in Table S1 in supporting information. Studies were not excluded based on their assessed quality.

### 2.4 Data extraction and synthesis

We developed two standardized data extraction forms, which were based on published meta‐ethnographic guidance.^20^ J.H. extracted descriptive data (study population, sample characteristics, country of origin, methods including data collection and synthesis) into one form. We additionally extracted information on study setting (routine clinical care, research setting, or a hypothetical/implementation context) to allow consideration of contextual influences on stakeholder perspectives. “Setting” was defined as the contextual use of the biomarker, rather than the physical location alone. For example, studies conducted in memory clinics were classified as research settings if biomarker testing or disclosure occurred as part of a research protocol rather than routine care.

Key findings, or “second‐order constructs” (interpretations made by the original authors), were independently extracted by J.H. and M.M. into a table in Microsoft Word, along with illustrative quotes from study participants (“first‐order constructs”). To maintain the context and meaning of the second‐order constructs, the original authors’ terminology and definitions were preserved. The completed forms were reviewed for consistency and discussed. Memo writing was conducted to maintain reflexivity. The review team included ten researchers: nine with clinical backgrounds and one with a non‐clinical background in behavioral science. All clinical members had experience in the assessment of patients with cognitive impairment, and four had experience of biomarker disclosure in clinical care or research settings. We were aware of our own professional biases and therefore we aimed to use a multi‐disciplinary approach, with both clinical (including psychiatrists, neurologists, and a psychologist) and a non‐clinical reviewer contributing to data analysis.

Study findings were synthesized using a meta‐ethnographic approach.^21^ Unlike traditional aggregative methods, meta‐ethnography emphasizes the development of analytical insights rather than just descriptive outcomes. It involves a process of “translation,” in which key concepts from one study are introduced into another and evaluated to determine how well they explain a particular phenomenon in different contexts.

Through this translation process, new interpretations, referred to as “third‐order constructs” emerge, providing a fresh understanding of the phenomenon under investigation. This was an iterative process and used the constant comparative method of qualitative synthesis.^22^ We aimed to generate constructs with high cogency, defined by agreement between reviewers of their clarity, impact, and underlying logic, that adequately reflect the primary data. We determined that the studies exhibited sufficient commonalities to support reciprocal translation, enabling us to juxtapose and synthesize key constructs across studies into cohesive third‐order constructs. When studies didn't agree, we analyzed why they differed to improve our findings (refutational translation), including both shared and conflicting ideas. To account for contextual variation, we considered study setting during meta‐ethnographic synthesis and examined whether the constructs differed across routine clinical, research, and hypothetical contexts.

Sub‐themes within each third‐order construct were then classified as representing barriers, enablers, or mixed enablers/barriers to AD biomarker use. We then deductively mapped these barriers and enablers to domains of the TDF that they were deemed to most closely represent. This provides a systematic and structured basis for the development of future behavior change interventions.

Finally, we created a “line of argument” synthesis, which integrates findings from all studies into a unified narrative, creating a new interpretation that explains varying perspectives on the use of AD investigations and the impact of identified barriers. This was possible as the patient, caregiver, and HCP studies did not directly contradict one another, but instead presented differing perspectives on AD biomarker investigations.

## 3 RESULTS

### 3.1 Search results

There were 4319 unique records identified after de‐duplication; 4244 of these were excluded after title and abstract screening. The remaining 75 full texts were assessed for eligibility and 49 records were excluded. Twenty‐six studies were included in the final review.

### 3.2 Characteristics of included studies

Characteristics of the included studies are presented in Table 2 and study‐level metadata in Table S1. Studies were conducted in 10 countries: United States (*n = *10), the Netherlands (*n *= 9), Belgium (*n = *2), UK (*n = *3), Canada (*n = *1), Germany (*n = *2), Sweden (*n = *1), China (*n = *1), Spain (*n = *1), and France (*n = *1). Sixteen studies used purely qualitative methodology and 10 used mixed methods. Six were conducted with HCPs; three with patients; three with caregivers; eight with patients and caregivers; and six with HCPs, caregivers, and patients. Studies were published between 2017 and 2024 and included a total of 2233 participants. This included HCPs (*n = *356), patients (*n = *855), caregivers (*n = *982), dementia care experts (*n = *3), healthy elderly (*n = *10), researchers (*n = *8), as well as one study which included health‐care funders (*n = *17) and members of advisory boards (*n = *2). HCPs included psychiatrists, neurologists, geriatricians, family or internal medicine providers, nurses, clinical psychologists, and primary care physicians.

**TABLE 2:** Characteristics of included studies.

Author (year, country)	Study aim and design	Data collection and analysis	Participants (*n*) and key demographics	Setting (*n*) and context^1^
Aspö^23^ (2024, Sweden)	To explore patients' perspectives regarding the diagnostic work‐up at a specialized memory clinic (observational, qualitative interview study)	Semi‐structured interviews with patients; content and thematic analysis	Patients (15); mean age 60.8 (range 50–72); 53.3% female; ethnicity NR; education: mean 14.6 years (range 11–19)	Specialized memory clinic (1) routine clinical care
Bélanger^24^ (2022, USA)	To examine care partners’ reactions to their loved ones receiving amyloid PET scan results (observational, mixed methods study)	Telephone survey with some open‐ended responses and a subset of audio recorded responses; content analysis	Care partners (196); age (years): < 65 (15.3%), 65–74 (47.4%), 75–84 (33.7%), ≥ 85 (NR); 68% female; 94.9% White, non‐Hispanic; education: high school or less (14.8%), some college (28.1%), college degree (22.5%), graduate degree (34.7%)	Dementia care practices and academic medical centers (415) research setting (CARE‐IDEAS cohort)
Bolsewig^25^ (2024, The Netherlands)	To evaluate informal caregivers’ attitudes toward undergoing and future implementation of BBM testing (observational, mixed methods attitudinal study)	Online survey with some open‐ended questions and a focus group; content and thematic analysis	Informal caregivers: survey (107); mean age 64.3 years (SD 11.4); 69.2% female; focus group (7); mean age 58.0 years (SD 9.9); 71.4% female; relation: 52.3% parent, 41.1% partner; race/ethnicity NR; education NR	Dutch patient organization community‐based hypothetical / implementation context
Couch^26^ (2022, USA)	To elucidate the relationship between amyloid PET scan results and subjective indicators of burden (observational, mixed methods study)	Telephone semi‐structured interviews; content and thematic analysis	Care partners (62); age years: < 65 (21.0%), 65–74 (51.6%), ≥75 (27.4%); 75.8% female; 54.8% non‐Hispanic White	Dementia care practices and academic medical centers (415) research setting (CARE‐IDEAS cohort)
Gadbois^27^ (2022, USA)	To examine perspectives and experiences of individuals with cognitive impairment who received an amyloid PET scan and their care partners (observational, mixed methods study)	Telephone semi‐structured interviews; inductive content analysis	Scan recipients (200) and care partners (200); recipients: age (years) 65–74 (52.0%), ≥ 75 (46.9%); 38.3% female; 95.4% White, non‐Hispanic; education: high school or less (16.8%), some college (24.0%), college degree (22.5%), post‐secondary (35.2%)	Dementia care practices and academic medical centers (415) research setting (CARE‐IDEAS cohort)
Grill^28^ (2017, USA)	To examine how amyloid PET imaging affects the diagnostic experience for patients and families (observational, qualitative study)	Telephone semi‐structured interviews; deductive analysis	26 patient–caregiver dyads; mean age 73.1 years (SD 10.3; range 52–88); 58% female; 87% White; mean education 17.2 years (SD 3.7)	Academic tertiary memory disorders clinic (1) routine clinical care (clinical amyloid imaging)
Hazan^10^ (2023, UK)	To explore clinicians' experience of the utility of a plasma biomarker test (observational, mixed methods)	Semi‐structured interviews; thematic analysis	Patients (29); mean age 74 years (SD 8.5); 65% female; diagnosis under assessment; education NR; ethnicity NR; 9 senior old age psychiatrists (8–35 years’ experience)	Memory services (2) routine clinical care
James^29^ (2020, USA)	To understand how accurately patients with MCI or dementia and their care partners report results of amyloid PET scans and factors related to correct reporting (observational, mixed methods)	Survey with some open‐ended question; content analysis	Patient–care partner dyads (202); patient age (CARE‐IDEAS cohort): MCI mean 74.6 years (SD 5.5), dementia mean 74.6 years (SD 5.8); care partner age mean years 70.1 (SD 9.6); 96.1% White; education: high school or less (14%), some college (28%), bachelor's degree (27%), graduate degree (31%)	Dementia care practices and academic medical centers (415) research setting (CARE‐IDEAS cohort)
Kim^30^ (2023, USA)	To describe the questions and concerns that may arise during the disclosure of AD biomarker results to persons with MCI and their family members (observational, qualitative analysis within experimental study)	Result disclosure sessions; content analysis	Patient–caregiver dyads (34); participants’ mean age 73.4 years (SD 8.0), care partners’ mean age 67.3 years (SD 11.2); 44.1% female; 85.3% White; education (participants): 58.8% graduate degree	Research Centre (1) University of Pittsburgh ADRC (RAISR study) research setting
Kunneman^31^ (2017, the Netherlands)	To explore clinicians’ views on and experiences with when, how, and by whom decisions about diagnostic testing for AD are made and how test results are discussed with patients (observational, qualitative study)	Focus group; thematic analysis	Neurologists (10), geriatricians (3); 54% female; affiliated with academic (*n =* 2), non‐academic teaching (*n =* 6), non‐teaching hospitals (*n =* 3), or mixed (*n =* 2); ethnicity NR	Dementia conference routine clinical care
Kunneman^32^ (2017, the Netherlands)	To assess patients’ and caregivers’ views on and experiences with decisions about diagnostic testing for AD and receiving test results (observational, qualitative study)	Focus groups; inductive and deductive content analysis	Patients (11); mean age 69 years (SD 6.6), 27.2% female; caregivers (11); mean age 64 years (SD 12.1), 72.7% female; education reported categorically (low/intermediate/high), defined as per study; distribution not summarized; ethnicity NR	Academic (1) or non‐academic hospital (2) routine clinical care
Linden^33^ (2024, the Netherlands)	To provide greater insight into the current decision‐making process on diagnostic testing for dementia (observational, qualitative study)	Semi‐structured interviews; inductive and deductive content analysis	Patients (20); mean age 72 years (range 63–82); 40% female; education: low (7), middle (7), high (6); significant others (15); mean age 69 years (range 50–82); 60% female; education: low (6), middle (4), high (5); primary care physicians (16) mean age 49 years (range 32–65), 45% female; practice nurses (2); mean age 63 years (range 59–68)	Primary care practices (unknown numbers) routine clinical care
Lingler^9^ (2018, USA)	To explore factors underlying decisions by patients with MCI to receive amyloid imaging results (observational, qualitative study embedded within RCT)	Semi‐structured interviews; content and thematic analysis	Patient–care partner dyads (MCI participants [30] & care partners [29]); patients’ mean age 72.9 years (SD 8.94), care partners’ mean age 68.2 years (SD 9.67); 63% female patients; 87% White; education: 80% > high school	Research center (1) University of Pittsburgh ADRC research setting
Lohmeyer^34^ (2021, Germany)	To explore and analyze participants' understanding and assessments of early detection and prediction of dementia in memory clinics (observational, qualitative study)	Semi‐structured interviews and focus groups; inductive and deductive content analysis	Patients (12): 41.7% female; 51–70 years (*n =* 5); > 70 years (*n =* 7); education: low secondary (3), intermediate secondary (5), high school (2), university (2); ethnicity NR. Caregivers (32): 84.4% female; education: low secondary (3), intermediate secondary (6), high school (4), university (11); age years 26–35 (*n =* 3); 36–50 (*n =* 2); 51–70 (*n =* 19); > 70 (*n =* 5); NR (*n =* 3); ethnicity NR	Community (4) and memory clinics (2) routine clinical care
O'Brien^7^ (2024, USA)	To identify and compare determinants of plasma AD biomarker use (observational, qualitative study)	Semi‐structured interviews; thematic analysis	Clinicians (30): family or internal medicine providers (16), geriatricians (8), and neurologists (6); reported (age categories in years: < 40 [10], 40–49 [5], 50–59 [6], 60–69 [7], ≥70 [2]); 53% female; ethnicity: 77% White, 23% Asian; 83% physicians, 17% nurse practitioners	Academic centers (2), Veterans Health Centre (1), and dementia clinical network (1) routine clinical care (implementation perspectives)
Patel^35^ (2024, Canada)	To describe patients’ and care partners’ experiences with AD CSF biomarker testing and result disclosure in routine care (observational, qualitative study)	Semi‐structured interviews; thematic content analysis	Patients (34): median age 63 years (IQR 56–68); 59% female; ethnicity: 86% White, 5.7% East Asian, 2.9% South Asian, 2.9% Indigenous; education: 82% post‐secondary; care partners (31): 58% female; age NR; education NR.	Specialist memory clinics (unknown numbers) routine clinical care
Smith^36^ (2024, USA)	To assess participants’ perspectives on the meaning of their amyloid PET imaging results (observational survey study)	Open‐ended survey responses; deductive analysis	Patients (88): mean age 71.6 years (SD 7.9); 45.5% female; 92% White/European American; 4.5% Hispanic/Latino; 1.1% Black; highly educated (74% bachelor's degree or higher)	Academic tertiary memory disorders clinic (1) routine clinical care (disclosure in a memory clinic, evaluated through a research follow‐up survey)
Suridjan^37^ (2023, USA, China, UK, Germany, Spain, France)	To explore heterogeneity in AD care pathways and potential role of BBM tests (qualitative; multi‐country survey study with advisory boards (market research design)	Survey and interview; thematic analysis	196 HCPs (PCPs, specialists including neurologists, geriatricians, psychiatrists), 17 payers and advisory boards (2); experience range: 3–35 years	HCPs: (unknown) Payers: Mix of private and public health care systems hypothetical/implementation context
Swallow^38^ (2020, UK)	To gather practitioners’ reflections on the utility and potential impact of prediction and earlier detection of AD (qualitative ethnographic study)	Ethnographic observations and semi‐structured interviews; thematic and situational analysis	HCPs (26) including memory nurses, trainee psychiatrists, consultant psychiatrists, clinical psychologists and consultant geriatricians	Memory clinics (2) routine clinical care
Tromp^39^ (2021, the Netherlands)	To explore the ethical considerations that shape current clinical practice regarding early AD diagnostics and the use of biomarkers (observational, qualitative study)	Semi‐structured interviews; directed content analysis	Physicians (15): PCPs (5), geriatricians (6), & neurologists (4); 40% female; 8–35 years clinical experience; mix of academic (*n =* 4 specialists) and community hospital (*n =* 6 specialists) settings	General practice, academic and community hospitals (unknown numbers) routine clinical care
van Gils^40^ (2022, the Netherlands)	To design a diagnostic report in co‐creation with patients and care partners (mixed‐methods co‐creation study)	Focus groups; directed content analysis	Patients (7); mean age 68 years, SD 6; 57% female; care partners (7); mean age 70 years, SD 4; 57% female; dementia experts (3); further demographics NR	Amsterdam Dementia Cohort, The Amsterdam Ageing Cohort & The Alzheimer's Society UK Routine clinical care
van Maurik^41^ (2019, the Netherlands)	To present the design, development, and testing of a Web‐based tool for clinicians in a memory clinic setting (iterative mixed‐methods co‐creation study)	Focus groups, panel discussion & interviews; thematic content analysis	Clinicians (13), patients (18), & caregivers (5); detailed demographics NR	Dementia conference (2) & memory clinics (4) routine clinical care
Vanderschaeghe^42^ (2017, Belgium)	To explore how patients with amnestic MCI perceive and experience amyloid PET scan disclosure in a research setting (qualitative study embedded in a diagnostic research trial)	Semi‐structured interviews; content analysis	Patients (38); mean age 70.9 years, SD 6.6; 42% female; mean education 13.2 years, SD 3.7 years; ethnicity NR	Memory clinic (1) research setting
Vanderschaeghe^43^ (2019, Belgium)	To explore stakeholders' views on early diagnosis and amyloid PET disclosure (observational, qualitative study)	Focus groups; content analysis	Healthy elderly (10); 80% female; education: primary‐professional bachelor; informal caregivers (9); 55% female; age years 40–75; education: secondary‐academic masters, nursing staff (6); 83% female; age years 18–70; researchers (8), 63% female; age years 30–70; clinicians (7), 100% male, age years 30–70	Social organization for the elderly, specialized dementia care home, pharmaceutical company, dementia organization research setting
Visser^44^ (2020, the Netherlands)	To examine uncertainty communicated by memory clinic clinicians in post‐diagnostic testing consultations with patients and their caregivers (observational mixed‐methods study)	Clinician–patient consultations; inductive and deductive content analysis	Patients (78): 41% female; mean age 70 years, SD 11; clinicians (22): 59% female; mean age 48 years, SD 10; neurologists (64%), geriatricians (36%).	Memory clinics (8) routine clinical care
Visser^45^ (2020, the Netherlands)	To explore clinicians’ communication, including the discussion of diagnosis, cause, prognosis, and care planning, in routine post‐diagnostic testing consultations with patients with MCI (observational qualitative study)	Clinician–patient consultations; thematic content analysis	Clinicians (10), neurologists (8), & geriatricians (2); mean age 49 years, SD 9; 40% female; mean memory clinic experience 10 years, SD 8. Patients (13): mean age 73 years, SD 9; 46% female	Memory clinics (7) routine clinical care

Abbreviations: AD, Alzheimer's disease; ADRC, Alzheimer's Disease Research Center; BBM, blood‐based biomarker; CSF, cerebrospinal fluid; HCP, health‐care professional; IQR, interquartile range; MCI, mild cognitive impairment; NR, not reported; PCP, primary care physician; PET, positron emission tomography; RCT, randomized, controlled trial; SD, standard deviation.

Ten studies took place in a memory clinic, six within research institutions, one was in a hospital, one was in general practice, three took place in the community (social organization or conference), and five were in a mix of these settings. When categorized according to the contextual use of biomarkers, sixteen studies reflected routine clinical practice, eight were conducted in research settings, and two examined biomarker use in hypothetical or health system planning scenarios.

### 3.3 Quality appraisal

The overall quality of included studies was moderate to high (Table 3). A common weakness was the lack of researcher reflexivity, meaning most studies did not adequately describe how the researchers’ backgrounds, assumptions, or relationships with participants might have influenced the findings. We assessed this using the CASP Qualitative Checklist, which evaluates whether studies discuss the researcher's role and potential biases.

**TABLE 3:** Quality appraisal of included studies.

First author (year of publication)	Clear statement	Qualitative appropriate	Research design	Sampling	Data collection	Reflexivity	Ethics	Data analysis	Discussion of findings	Value	Overall assessment of methodological quality
Aspö 2024	Y	Y	Y	Y	Y	N	Y	Y	Y	Y	Moderate‐to‐high
Bélanger 2022	Y	Y	Y	Y	N	N	Y	Y	Y	Y	Moderate‐to‐high
Bolsewig 2024	Y	Y	Y	Y	Y	N	Y	Y	Y	Y	Moderate‐to‐high
Couch 2022	Y	Y	Y	Y	Y	?	Y	Y	Y	Y	Moderate‐to‐high
Gadbois 2022	Y	Y	Y	Y	Y	N	Y	Y	Y	Y	Moderate‐to‐high
Grill 2017	Y	Y	N	N	Y	N	Y	Y	Y	Y	Moderate‐to‐high
Hazan 2023	Y	Y	Y	Y	N	N	Y	N	Y	Y	Moderate‐to‐high
James 2020	Y	Y	Y	?	Y	N	Y	Y	Y	Y	Moderate‐to‐high
Kim 2023	Y	Y	N	Y	Y	N	Y	Y	Y	Y	Moderate‐to‐high
Kunneman 2017	Y	Y	Y	?	Y	N	N	?	Y	Y	Moderate
Kunneman 2017	Y	Y	Y	Y	Y	N	Y	Y	Y	Y	Moderate‐to‐high
Linden 2024	Y	Y	Y	Y	Y	Y	Y	Y	Y	Y	High
Lingler 2018	Y	Y	Y	Y	Y	N	Y	Y	Y	Y	Moderate‐to‐high
Lohmeyer 2021	Y	Y	Y	Y	Y	N	Y	Y	Y	Y	Moderate‐to‐high
O'Brien 2024	Y	Y	Y	Y	Y	Y	Y	Y	Y	Y	High
Patel 2024	Y	Y	Y	Y	Y	N	Y	Y	Y	Y	Moderate‐to‐high
Smith 2024	Y	Y	Y	Y	N	N	Y	Y	Y	Y	Moderate‐to‐high
Suridjan 2023	Y	Y	Y	?	?	N	Y	?	Y	Y	Moderate
Swallow 2020	Y	Y	Y	Y	Y	N	Y	Y	Y	Y	Moderate‐to‐high
Tromp 2021	Y	Y	Y	Y	Y	N	Y	Y	Y	Y	Moderate‐to‐high
van Gils 2022	Y	Y	Y	Y	Y	N	Y	Y	Y	Y	Moderate‐to‐high
van Maurik 2019	Y	Y	Y	Y	?	N	Y	?	Y	Y	Moderate‐to‐high
Vanderschaeghe 2017	Y	Y	Y	Y	Y	N	Y	Y	Y	Y	Moderate‐to‐high
Vanderschaeghe 2019	Y	Y	Y	N	Y	N	Y	Y	Y	Y	Moderate‐to‐high
Visser 2019	Y	Y	Y	Y	Y	N	Y	Y	Y	Y	Moderate‐to‐high
Visser 2020	Y	Y	Y	Y	Y	N	Y	Y	Y	Y	Moderate‐to‐high

Abbreviations: N, no or not sound methodology; Y, yes or sound methodology;?, can't tell if there is sound methodology.

### 3.4 Translation

Through translation, we synthesized second‐order constructs from included primary studies into third‐order constructs, as illustrated in Table S3 in supporting information, which maps the first‐, second‐, and third‐order constructs. The review synthesized 19 third‐order constructs, grouped into five key concepts. These constructs, summarized in Table 4, were mapped to their corresponding barriers, enablers, TDF domains, and study setting. Table S4 in supporting information provides supporting illustrative quotes for these barriers and enablers.

**TABLE 4:** Summary of qualitative findings mapped to the TDF domains and barriers and enablers for AD biomarker testing.

Third‐order construct	Barrier/enabler/mixed	TDF domain(s)	Context (Research settting, routine care or hypothetical implementation)
**Concept 1: Expectations and motivations of stakeholders**			
Caregivers and patients place different value on the need for biomarker testing.	Caregivers seek out testing (25) in cases where patients can lack the motivation to do so (26,27) (M)	Intentions	HI, RS
	Caregivers encourage patients to seek testing (27,32) which can lead to tension between stakeholders (34) (E)	Social influences, emotion	RS, RC
If caregivers and patients decide to seek biomarker testing, their motivations may differ.	Patients seek testing to gain information for “knowledge's sake” (9) and caregivers use it to validate their experiences (34) (E)	Goals	RS, RC
	Caregivers use testing to inform care and treatment planning (28)(E)	Behavioral regulation	RC
	Caregivers and patients perceive testing will lead to both positive outcomes (such as treatment access [27,30], clarity of symptoms and a sense of control [33], and negative outcomes [25,30,36]) (M)	Beliefs about consequences	RS, RC, HI
Satisfaction with testing depends on how well participants’ expectations matched with the reality of what testing can provide.	Patients and caregivers have expectations that symptoms are due to AD or dementia (28) (E)	Knowledge	RC
	Patients have high expectations for testing (23,27) and can express a sense of frustration if these are not met (23) (B)	Beliefs about consequences, emotion	RS, RC
Prior experiences and personal context shape the desire for biomarker testing.	Patients and caregivers seek out a doctor's appointment based on their personal experiences of AD in the family (27) (E)	Intentions	RS
**Concept 2: Attached meaning of the test result**			
Willingness to undergo testing is shaped by perceptions of perceived availability or unavailability of treatments or interventions.	Caregivers are optimistic testing will enable access to care and treatment options (24) (E) Both patients and HCPs vary in the degree to which they value the need for testing and this is related to their optimism or pessimism regarding treatments (7,25) (M)	Optimism	RS, RC, HI
	Patients who lack knowledge about the limitations of available treatments are more likely to see the need for testing (30) (E)	Knowledge, beliefs about consequences	RS
	Some stakeholders value early diagnosis as this is perceived as facilitating early treatment (34) (E)	Beliefs about consequences	RC
	Stakeholders raise ethical concerns with testing in the absence of disease modifying treatments (34) (B)	Social professional role & identity	RC
Psychosocial impacts vary between groups and from relief to consideration of suicide.	Some patients and caregivers experience hope and acceptance after a positive test result (24,28), whereas others experience despair (24) (M) Caregivers and HCPs express concern over the anticipated emotional burden of a test result for the patient (7,9) while patients express the same for their family (30) (B)	Emotion	RS RC, RS
	Optimistic appraisals of a positive result may spur adoption of health‐positive behaviors (23) and coping strategies (26), whereas pessimistic appraisals may lead to suicidal ideation as an act of self‐determination (34,36) (M)	Optimism, Memory, attention & decision processes	RC, RS
All stakeholders perceive the possibility of stigma attached to a test result.	All stakeholders are concerned with stigma associated with a test result (7,26,30,34,43) (B)	Social influences, social professional role & identity	RC, RS
The diagnostic and prognostic value placed on the test result by patients and caregivers does not always align with the information that the test provides.	Lack of knowledge of risk/prognostication (30) (B)	Knowledge	RS
There is no consensus among HCPs on where AD is defined along a clinical to biological continuum.	Variability in knowledge of AD definition (39) (B)	Knowledge	RC
	Some HCPs question the value of biomarker testing as they perceive AD as a purely clinical diagnosis (39) while others express a biomarker test will enhance diagnostic accuracy (10) (M)	Beliefs about consequences	RC
**Concept 3: Shared decision‐making and result communication**			
The decision to offer biomarker testing is typically made by HCPs, with shared input from patients and caregivers being more aspirational than commonly practiced.	Some HCPs question the value of biomarker testing in patients with advanced age (7,39) (B) HCPs are concerned about the potential negative impact of providing a diagnosis too early (7) (B)	Beliefs about consequences	RC
	Shared decision‐making is important to all stakeholders, but HCPs may use a more directive approach, limiting true patient involvement (32,33) (M)	Social professional role and identity, memory, attention & decision processes	RC
	Some primary care physician referrals to memory services include an implicit endorsement of biomarker testing (32) while dementia specialist HCPs’ language can explicitly convey a dismissive attitude toward biomarker testing (44) (M)	Social influences, memory, attention & decision processes	RC
Pre‐test counseling involves limited focus on the potential negatives of testing and patients may instead appraise the decision to test in the context of prior experiences of medical care.	Patients find the decision to undergo testing straightforward (9), in part due to a lack of knowledge about test rationale and implications (30,32) (E) Patients found the decision to test comparable to past health‐care decisions while caregivers found it more complex than past health‐care decisions (9) (M)	Memory, attention & decision processes, knowledge	RS, RC
	Patients and caregivers have trust in HCPs’ decision to suggest testing (27,32,33) (E)	Social influences	RS, RC
Adequate test result communication is a vital but challenging component of testing.	HCPs express a lack of knowledge in interpreting the result (7,30) (B)	Knowledge	RC, RS
	A printout summary report or visual representation of the biomarker result is valuable for patients and caregivers (10,27,30) (E)	Memory, attention & decision processes	RC, RS
	Caregivers value having time to discuss results with other members of the multidisciplinary team, although in practice they often lack of time to discuss the test result (32) (M)	Environmental context & resources	RC
	HCPs provide inadequate education on the differences between normal aging, MCI (9,27,34), and symptoms of dementia (27) (B)	Knowledge	RS, RC
	A lack of knowledge regarding what the test measures (9,28,30) may lead patients to understand and report their test result inaccurately (29) (B)	Knowledge	RC, RS
	HCPs can use confusing language (30) when communicating the test result and may benefit from training (7) (M)	Skills	RS, RC
**Concept 4: Desire for diagnostic certainty**			
Participants express a desire to undergo testing as a means of providing diagnostic certainty.	Stakeholders request biomarker testing to remove uncertainty (7,27) sometimes by reducing reliance on exclusion‐based diagnosis (7) (E)	Beliefs about consequences	RC, RS
	Patients express dissatisfaction with testing when it does not provide diagnostic certainty (34) (B)	Emotion	RC
Greater diagnostic weighting is placed on biomarker tests when they are invasive whereas this is reduced if the result conflicts with the clinical impression or other test results.	Difficulties can arise when the test result conflicts with the clinical impression, leading some HCPs to prioritize the clinical and potentially reducing their reliance on test findings in decision‐making (10) (B)	Beliefs about consequences, memory, attention & decision processes	RC
	Visible biomarker evidence strengthens trust in diagnosis and supports decision‐making (28,32) (E)	Beliefs about consequences	RC
**Concept 5: systems and pathways to test delivery**			
There is variability in perceived accessibility of biomarker testing between stakeholder groups.	There is limited accessibility of testing (6) and of specialist assessment (27,37) (E)	Environmental context & resources	RC, RS, HI
Variability in primary care physician referrals to memory services is influenced by the primary care physician's clinical skill and knowledge.	Lack of knowledge of the evidence base for the test (27) (B)	Knowledge	RS
	Lack of training in recognizing disease symptoms and assessing cognitive complaints in primary care (27) (B)	Skills	RS
	There is variable ease in accessing a referral to specialist services (27) (M)	Environmental context & resources	RS
HCPs’ willingness to use a biomarker test is impacted by the test properties.	Blood biomarker tests are less resource intensive in terms of time to perform the test and cost (37) (E)	Environmental context & resources	HI
	The high positive predictive value of the test provides greater confidence in results, enhancing clarity and certainty in diagnosing AD (37) (E)	Belief about consequences	HI
	More invasive biomarker tests are less acceptable to some HCPs (39) (B)	Memory, attention & decision processes	RC
HCPs use biomarker tests in idiosyncratic ways.	HCPs differ in their approach to when to order biomarker testing in relation to other investigations (7) (M)	Memory, attention & decision processes	RC
	HCPs are unaware of guidelines (39) for use and there is a need for clear guidelines on biomarker use (7) (M)	Knowledge	RC
	Non‐specialist HCPs may misuse test result information (7) (B)	Beliefs about capabilities, Social professional role & identity	RC
	HCPs are more likely to test if there is confirmation from peer or authority influences (7) (E)	Social influences, Social professional role & identity	RC

Abbreviations: AD, Alzheimer's disease; B, barrier; E, enabler; HCP, health‐care professional; HI, hypothetical implementation; MCI, mild cognitive impairment; M, mixed; RS, research setting; RC, routine care; TDF, theoretical domains framework.

#### 3.4.1 Expectations and motivations of stakeholders

This concept comprised four third‐order constructs. The first described varying stakeholder perspectives on the need for testing, diverse stakeholder motivations for biomarker testing, the influence of expectations on satisfaction with testing, and how personal history shapes the desire for biomarker testing.

Caregivers were often the “driving force” behind instigating testing, which raised ethical concerns about patient autonomy, the right to choose, and the burden of pressure.^23^, ^24^, ^25^ This also created tension, as patients may not have identified this need,^24^ leading to conflict with caregivers and in one instance a caregiver becoming “the hated daughter.”^23^

Patients and caregivers had diverse expectations and motivations for pursuing testing.^23^ Caregivers perceived that testing would enable future planning,^9^, ^23^, ^26^, ^27^, ^28^, ^29^, ^30^, ^31^ access to specialist services,^24^, ^29^ and self‐management tools,^32^ whereas a primary motivation for patients was to seek answers and clarity regarding the cause of their symptoms.^9^, ^23^, ^25^, ^27^, ^28^ This desire for information for its own sake stemmed from a need to understand what is happening^9^, ^27^, ^28^, ^29^, ^30^, ^33^, ^34^ and gain a sense of control^24^, ^30^ and was independent of need to establish their diagnosis.^9^

Satisfaction with testing was related to how well participants’ expectations matched with the reality of what testing could offer.^33^, ^35^ When participants sought a diagnosis or treatment, but were left without one, they had a more negative appraisal of testing and expressed frustration.

Personal experiences and individual context shape the desire for biomarker testing. For example, patients and caregivers with a family history of AD, or those who have seen its impact within their social circles, may feel a stronger motivation to pursue testing.^23^, ^27^, ^34^

#### 3.4.2 Attached meaning of the test result

This concept encompassed six third‐order constructs: willingness to undergo testing, psychosocial impacts of test results, stigma, lack of understanding about what the test measures, varying definitions of AD, and caregivers’ use of test results to develop coping strategies.

Willingness to undergo testing was shaped by perceptions of available treatments.^7^, ^9^, ^23^, ^24^, ^25^, ^26^, ^27^, ^28^, ^31^, ^34^, ^35^, ^36^, ^37^, ^38^, ^39^, ^40^ Some individuals viewed testing as a pathway to future therapies, especially with advancements in disease‐modifying treatments (DMTs),^7^, ^34^, ^38^ while others questioned the ethics of disclosing results in the absence of such treatments.^7^, ^24^, ^38^ Caregivers^23^, ^24^ and specialist HCPs emphasized the importance of early diagnosis,^38^ though primary care physicians were less convinced of its benefits.^7^, ^34^ Some patients viewed the test result as a catalyst for adopting healthier behaviors, making lifestyle changes, and cultivating a sense of self‐efficacy.^24^, ^25^, ^30^, ^33^

Some HCPs believed biomarkers had little value because an AD diagnosis for them is based largely on clinical symptoms, while others saw it as biologically defined.^37^, ^40^, ^41^ Patients and caregivers sometimes equated a positive biomarker result with a definitive AD diagnosis.^9^, ^28^ Some participants expressed belief that there was a direct correlation between the amount or extent of amyloid plaque seen on a scan and disease severity, though HCPs advised against using amyloid imaging for this purpose.^9^, ^28^

Test results elicited a range of emotions.^29^ One study found that most patients and caregivers feel positive after receiving biomarker test results.^30^ Caregivers often felt sadness or despair after a dementia diagnosis, though some experienced relief or comfort in having clarity; for patients, however, this sense of relief was generally more limited. Test results brought a relief and validation to some caregivers, confirmed their suspicions and facilitated open discussion of the diagnosis.^23^, ^24^, ^27^, ^28^ Caregivers' roles were influenced by the test results, the identification of AD pathology was used to develop coping strategies,^32^ while those left without clear answers struggled with unexplained cognitive decline.

Both caregivers and HCPs expressed concerns about whether testing was in the patient's best interest, given the potential for stigma^24^ and heightened emotional burden.^24^ Patients, too, were worried about the stigma associated with a test result and feared that it might burden their families,^29^, ^30^ whereas family members valued the information as a means to better support the patient. One patient described the diagnosis as a “verdict,” expressing fear of the future; others discussed suicide or euthanasia as an assertion of self‐determination.^24^

#### 3.4.3 Shared decision‐making and test communication

This concept comprised three third‐order constructs: restricted shared decision‐making, limited pre‐test counseling, and the significance and challenges of effective test result communication.

Patients, caregivers, and HCPs brought their different preferences to the decision‐making process. Patient characteristics, such as advanced age, affected HCPs’ decisions to refer for or conduct testing, with advanced age being cited as a barrier to testing. Some patients appreciated clinicians making decisions on their behalf, while others felt excluded and questioned whether they received optimal care.^23^ Many patients and caregivers felt involved in decision‐making,^23^ but often lacked crucial information about the pros and cons of testing, limiting the extent of participation in shared decision‐making.^41^ This information gap raised ethical concerns in some studies.^41^ Patients and caregivers tended to focus on the benefits of testing,^9^, ^23^, ^24^, ^28^ with patients expressing that it was an “easy” decision.^9^, ^30^ Most patients expressed that they had not regretted the decision to have testing.^28^ Given the complexity of these results, often involving probabilities and uncertainties, stakeholders valued image and text resources as aids to understanding their test result.^28^, ^29^

Studies described a lack of knowledge about test interpretation and diagnostic terminology, as well as insufficient skills in communicating these concepts to patients. Both patients and caregivers hoped the test result would provide prognostic information^9^, ^23^, ^24^, ^29^ and some lacked awareness of the limitations of the test result information.^41^ There was confusion between diagnostic terms like MCI and dementia,^29^ as well as uncertainty about what biomarker tests measure and their limitations.^9^, ^28^, ^29^ This uncertainty was more pronounced in those with an MCI diagnosis and their caregivers, who struggled to understand the early stages of AD and the significance of biomarker results.^24^, ^41^

HCPs struggled to communicate test‐related uncertainty. One study highlighted the need for better pre‐counseling to manage expectations about the diagnostic confidence biomarker testing can provide.^28^ HCPs often addressed the cause of symptoms tentatively, which did not fully meet the patients’ and caregivers’ needs for clarity. Several studies raised concern over HCPs’ ability to accurately interpret results, highlighting the potential for misinterpretation.^7^, ^10^ This included the challenge of interpreting results along a continuum, rather than as a binary outcome.29

Additionally, there was inconsistency in how clinicians explained MCI, leading to varying patient understanding.^24^, ^29^ Participants had differing opinions on how a pathological process, as defined by results of biomarker testing, differed from normal aging.^27^ The use of medical jargon or ambiguity led to miscommunication of test result meaning, with some patients and caregivers inaccurately reporting their result.^29^, ^41^ For instance, some participants mistakenly perceived a “positive” biomarker test result as indicating a favorable outcome, with implication of absence of disease pathology.^29^, ^41^

#### 3.4.4 Desire for diagnostic certainty

This concept included two third‐order constructs which described: (1) a desire to reduce diagnostic uncertainty by undergoing testing, and (2) factors influencing the relative weighting of the test result in the overall diagnosis. An enabler to testing for participants, especially caregivers, was the expressed strong desire for diagnostic certainty to remove ongoing uncertainty and enable informed decision‐making.^23^, ^30^ However, when test results did not provide a clear diagnosis or explanation for cognitive symptoms, this acted as a barrier, with participants reporting frustration, disappointment, and anxiety. A lack of personalized advice, treatment, and follow‐up left many uncertain about the future.

Some HCPs found biomarker test results increased diagnostic certainty, while others found the result increased ambiguity and introduced conflict, leading them to prioritize the clinical presentation in determining diagnosis.^10^

Some patients and caregivers felt that more invasive investigations, such as lumbar punctures, added a “physicality” and thereby greater certainty and reality to the test result.^24^, ^28^, ^30^

#### 3.4.5 Systems and pathways

This concept encompassed four third‐order constructs: variability in access to biomarker testing, the influence of general practitioners’ clinical skills and knowledge on memory service referrals, the impact of test characteristics on willingness to use the test, and the idiosyncratic application of the test by HCPs.

Access to AD biomarker testing was variable among stakeholders, with barriers such as insurance coverage gaps^28^ and logistical delays limiting availability in some health‐care systems. Caregivers more frequently raised concern over long wait times compared to patients,^27^ though perceptions of the accessibility process varied. Several studies mentioned caregivers engaging in multiple consultations to convince HCPs of symptoms.^27^ Demand for testing frequently outpaced supply due to inefficiencies and the absence of streamlined pathways to testing. Patients and caregivers cited concerns such as the cost, time, and inconvenience,^28^ while HCPs favored tests which were time efficient and scalable.^38^

Variability in HCP practices also extended to primary care physicians, whose referrals to memory services were influenced by their clinical skills and familiarity with cognitive assessment and biomarker testing.^27^ Limited awareness among primary care physicians resulted in certain biomarker testing options not being discussed with patients.^27^ Studies highlighted the importance of primary care physicians as the gatekeepers to access for testing.^23^

Stakeholders’ willingness to use biomarker tests often depended on factors such as invasiveness^40^ and diagnostic accuracy.^38^ Clinicians sometimes lacked knowledge in appropriate use recommendations and cited guidelines produced by trusted experts as an enabler to biomarker testing.^7^

#### 3.4.6 TDF analysis

In total, 18 barriers, 17 enablers, and 15 mixed barrier/enabler relevant to AD biomarker test use were identified. These findings, along with their corresponding TDF domains and participant quotations, are detailed in Table S3 and summarized in the following text, with the corresponding TDF domains in parentheses italicized.

The most common mixed barriers/enablers related to support in shared decision‐making, perceived complexity of the decision to test by patients and caregivers, and the language HCPs use to endorse the test (*memory, attention*, and *decision processes*). However, communication tools were a common enabler to support test use (*memory, attention*, and *decision processes*). Other frequent enablers included patients’ trust in HCPs’ recommendation to test or HCPs’ trust in recommendations by peers (*social influences*). The most common barriers to AD biomarker testing related to lack of knowledge about biomarkers (*knowledge*) and experience of emotional burden after testing (*emotion*). “Beliefs about consequences” described a similar number of barriers and enablers related to the perceived pros and cons of testing. One domain, “reinforcement,” yielded no barriers or enablers, potentially because the included studies did not include statements regarding HCPs’ incentives or sanctions to repeating testing.

#### 3.4.7 Comparison of findings across study settings

Findings related to the perceived value of testing, HCP practices, and diagnostic certainty were primarily identified in routine clinical care settings, whereas testing in the context of treatment optimism emerged predominantly in research settings. In contrast, findings related to knowledge of biomarker testing, concerns about stigma, and emotional responses were evident across both routine and research settings. System‐level implementation barriers and findings related to test properties emerged primarily in hypothetical or health‐system planning contexts.

### 3.5 Line of argument synthesis

The syntheses of studies revealed that differences exist among stakeholders with differing priorities and perspectives. Patients and caregivers have differing motivations and intentions to pursue testing. Primary care physician barriers in knowledge and skill related to AD testing may prevent patients being referred for testing in the first place (*knowledge*). Therapeutic pessimism of HCPs acts as a barrier to testing. All stakeholders are concerned about negative consequences of testing, with unintended psychosocial consequences (*beliefs about consequences*). Indeed, some patients and caregivers face a negative emotional burden of testing (*emotion*). Trust in the opinions of doctors or peers was a significant enabler for the use of biomarker testing (*social influences*).

Patients may face epistemic injustice, with HCPs not providing sufficient information to enable them to make fully informed decisions about the reasons for testing or the associated risks and benefits. Lack of resources or clear pathways may limit implementation of testing.

**FIGURE 2:**
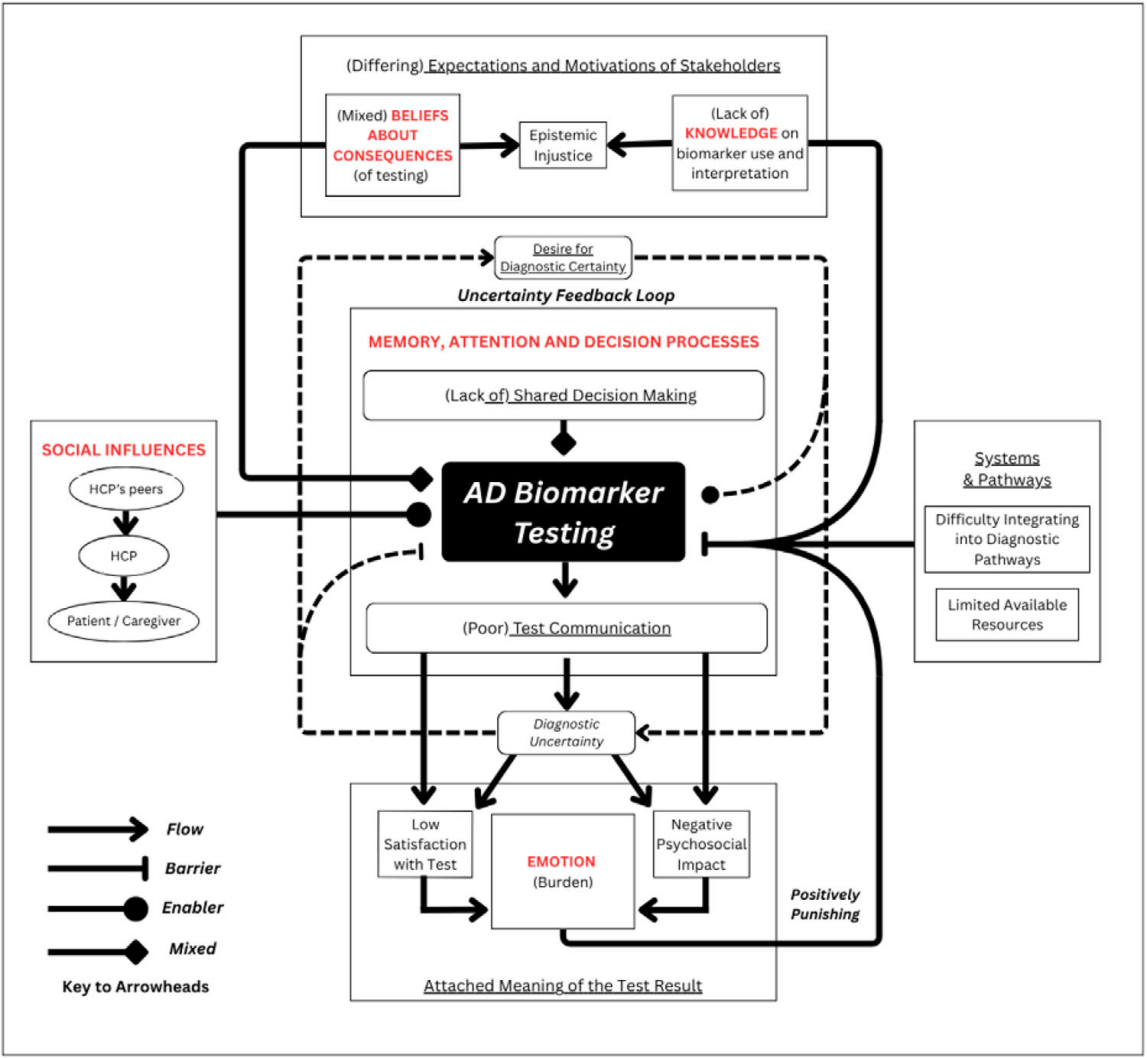
The line of argument synthesis demonstrates how the key concepts and salient TDF domains intersect to provide a unified explanation of the perceptions and behaviors surrounding AD biomarker testing. Key concepts are underlined. TDF domains are in red typeface. Arrows represent relationships and flow between concepts: T arrowheads represent barriers to test use. Circle arrowheads represent enablers to testing. Diamond arrowheads represent mixed barriers/enablers. Dashed arrow represents an uncertainty feedback loop. AD, Alzheimer's disease; HCP, health‐care professional; TDF, theoretical domains framework.

For HCPs, deciding whether to test and how to communicate the results are challenging cognitive processes (*memory, attention*, and *decision processes*). Patients and caregivers perceive that testing will provide diagnostic certainty. Testing, intended as a tool to manage uncertainty, can paradoxically introduce further uncertainty, creating an “uncertainty feedback loop,” in which efforts to clarify lead to additional questions or doubts. This cycle underscores the challenges of test interpretation and of balancing transparency with reassurance in the face of complex and evolving diagnostic landscapes. Satisfaction with testing outcomes is influenced by how uncertainty is communicated: if HCPs acknowledge the unknowns, patients and caregivers may initially feel frustrated but are better prepared for nuanced results. Conversely, if uncertainty is downplayed, they may experience confusion or disappointment later, potentially leading to misinterpretation of results. This unified interpretation is presented in Figure 2.

## 4 CONCLUSIONS

This systematic review is the first to synthesize qualitative evidence on perceptions of biomarker investigations for AD. The meta‐ethnographic synthesis revealed a landscape describable in terms of opposites: between clinical and biological definitions of AD, between certainty and uncertainty in results, between empowerment and disempowerment of patients, and between accessibility and inaccessibility to testing.

The lack of consensus we identified in how AD is defined along the biological to clinical continuum reflects the recent conflicting diagnostic recommendations.^4^, ^42^ Biomarker tests attempt to formalize the diagnosis in biological terms, and may improve accuracy of subtyping dementia diagnoses.^42^ However, depending on what one considers to be the gold standard definition, this can introduce a “category error” by equating biomarker positivity with disease,^43^ further risking conceptually invalid test use (e.g., in using the results to prognosticate or in individuals without objective impairments) and inconsistent test interpretation when faced with conflicting or indeterminate results.^44^ This has the potential to cause iatrogenic harm.

Quantitative systematic reviews have predominantly focused on the clinical utility of AD biomarker testing, such as its effects on diagnostic confidence and access to AD management.^6^ While biomarkers can enhance diagnostic confidence, inconsistencies may arise due to differences in clinician training, decision‐making strategies, and experience with complex cases.^6^ This is hypothesized by our uncertainty feedback loop influencing testing decisions. This feedback loop may perpetuate hesitancy and variability in testing practices. Studies also reveal challenges in reconciling clinical presentations with biomarker findings, leading to uncertainty and variability in diagnostic approaches.^45^ Addressing these issues may require standardized training and clearer guidelines to support clinicians in effectively interpreting biomarker results. Such training could include guidance on communicating probabilistic risk and uncertainty, strategies for managing expectations on what biomarker test information provides, and case‐based scenarios to support decision making in situations such as when there is non‐concordance between the clinical presentation and biomarker result.

Increased diagnostic uncertainty after testing recurs as a prominent negative consequence, though it appears to be rarely anticipated by any stakeholder party, possibly reflective of bias toward the putative gains of testing.^36^ While HCPs experienced in using these tests will know that the results are not binary—that is, positive/negative—this is not always known by less experienced clinicians and is not always well communicated to patients.^46^ The effect of receiving an uncertain result has been variously described as placing an individual “in a liminal state between health and disease,”^47^ and as “patients‐in‐waiting,”^48^ emphasizing its harmful sociological aspects. This may reinforce the notion of being subject to an unknown and unpredictable disease process.^49^

Disempowerment can also follow a positive test result, though this may also drive the adoption of positive reflective attitudes and self‐determining behaviors.^33^ This phenomenon has been demonstrated in the medical literature, and it is not clear how to predict how an individual patient will respond to their test result.^50^ The possibility of suicidal ideation (specified as an act of self‐determination) after testing is an important consideration when pre‐counseling patients to have the test, given that there is a heightened risk of suicide after dementia diagnosis.^51^

Accessibility to testing can be considered across multiple levels. Previous studies have highlighted geographic variation in service availability and access to limited resources as a barrier to accessing specialist AD investigations.^52^ A patient's demographic factors, for example, socioeconomic deprivation and minority ethnic status, have been associated with reduced dementia diagnosis rates.^53^ A patient's cognitive impairment may also present barriers to navigating access to testing (e.g., difficulties in self‐advocacy).^54^ This leads to uneven parity in diagnosis; a biomarker‐supported AD diagnosis is not conceptually equivalent to one made on clinical grounds alone.

### 4.1 Limitations

This review has several limitations. First, it is possible that not all relevant studies were captured and the quality and scope of included studies constrained our findings. Most studies did not specifically focus on identifying barriers and enablers to AD biomarker testing, with only two studies addressing this explicitly, one of which was the index paper.^7^, ^38^ This may have limited the depth of insights obtained. Furthermore, distinguishing between patient and caregiver perspectives proved challenging, as their data were often combined, reducing the specificity of our analysis.

Although the majority of included studies reflected routine clinical care, many of these were conducted in academic centers, as this is where most AD biomarker investigations have taken place to date. While this focus reflects current practices, it may limit the applicability of findings to community‐based settings. There may be different attitudes to plasma biomarker testing, which has only recently become available.

Eight studies were undertaken in research or trial‐linked settings in the context of a research protocol. However, these studies were performed in the context of a diagnostic evaluation, as opposed to trial recruitment or asymptomatic screening. To increase comparability across settings, we limited participant inclusion to individuals with cognitive impairment, rather than asymptomatic participants, as biomarker testing in this context carries similar diagnostic implications in both research and clinical settings. However, it is important to consider that the contextual setting may have influenced our findings. Research environments are more controlled, allow greater time for patient counseling, follow strict protocols, and may not encounter the same resource constraints as routine clinical practice.

Additionally, differences in health‐care systems across regions reduce the generalizability of system‐level barriers, as financial models and care pathways vary widely. Finally, this review included only studies conducted in English and in Western countries, so the findings largely represent the views of participants within specific cultural contexts. Where reported, the participants were predominantly White and had relatively high levels of education attainment. However, reporting of ethnicity and education attainment was inconsistent across included studies, with several studies not providing this information in detail.

The authors were cognizant that their clinical backgrounds, professional interests, and experience with biomarker testing could shape their interpretation of the findings. To address this, reflexive memo writing was maintained and authors discussed how their practice and position may influence the synthesis of findings. The inclusion of five researchers without experience of the use of diagnostic biomarker testing helped to ensure a diversity of experience and viewpoints.

### 4.2 Implications for future research, practice, and policy

Our review aimed to identify and integrate factors influencing testing. By moving beyond individual study findings, this review contributes a more holistic understanding of the determinants of AD biomarker testing. Our findings highlighted varied stakeholder perspectives on AD biomarker testing. Accounting for these differences and engaging all relevant stakeholders in the design and implementation of future interventions is essential to ensure their acceptability and sustain long‐term change.^55^

We mapped key constructs to the TDF, identifying barriers and enablers to testing to inform the design of effective implementation interventions. By aligning constructs such as trust in HCPs, variability in access, and emotional burden with TDF domains (e.g., *social influences, environmental context and resources, emotion*), we identified actionable targets to design theory‐informed interventions and policies to optimize AD biomarker testing adoption.

For example, studies highlight that HCPs often lack sufficient understanding of biomarker testing, either leading to absence of use, or misuse of the test.^56^ This is a particular concern for use by non‐specialists. Policy makers could prioritize evidence‐based training programs and national guidelines to address these knowledge gaps. These could also include communication frameworks to help HCPs structure sensitive discussions^57^ and deepen their understanding of the potential emotional impact on patients and caregivers.^58^ Studies assessing HCPs’ appraisal of their own knowledge or skill gaps in the AD biomarker field are limited and this warrants further exploration, particularly as blood biomarker testing will likely rapidly expand access in community settings where they have previously been unavailable.

Training programs should also proactively address HCPs’ concerns about potential negative outcomes, while clearly highlighting the benefits of testing, with stakeholder testimonials integrated to effectively illustrate these advantages. Intervention strategies should promote the development of decision‐making tools^59^ and communication aids^60^ to boost HCP confidence in facilitating shared decision‐making.

Policies to implement AD biomarker testing into routine practice must factor in adequate resources and establish clear pathways for test use. The relatively low expense of blood‐based biomarkers (BBM) make them attractive for wide‐scale use, though only four of the included studies focused on AD BBMs;^7^, ^10^, ^36^, ^38^ future studies should collect data to specifically address this. A recently published qualitative study of Dutch general practitioners’ perspectives on BBMs similarly identified gaps in clinician knowledge, mixed beliefs about the consequences of testing, and certainty regarding diagnosis, reinforcing our findings regarding variability in clinician practice and training needs. AD BBMs may also face fewer barriers related to skills in sample collection, and test acceptability compared to currently available investigations. TDF‐based studies specifically characterizing such barriers and enablers could inform the prioritization of intervention designs.

AD biomarker testing in clinical practice is a complex process, with differing stakeholder perspectives. To improve their use and support the implementation of AD blood biomarkers, which are already being used in some clinical settings, it is essential to understand how these factors interplay and to design targeted interventions and policy that address identified barriers and enablers. These may include evidence‐based training, national guidelines, structured communication frameworks, decision‐support tools, and the establishment of clear community phlebotomy pathways.

## CONFLICT OF INTEREST STATEMENT

The authors declare that they have no conflicts of interest. Author disclosures are available in the Supporting Information.

## ETHICS APPROVAL AND CONSENT TO PARTICIPATE

Not applicable. This study was a systematic review based exclusively on previously published data and did not involve direct participation of human subjects.

## Supporting information




**Supporting Information**: dad270380‐sup‐0001‐SupMat.docx

**Supporting Information**: dad270380‐sup‐0002‐ICMJE.pdf

## Data Availability

All data generated or analyzed during this study are included in this published article and its supporting information files.
