# Supplementary material for: Perceptions of the use of biomarkers for Alzheimer's disease diagnosis: A systematic review and synthesis of the qualitative literature
Source: Alzheimers Dement (Amst). 2026 Jun 3;18(2):e70380. doi: 10.1002/dad2.70380 (PMC13239579; doi:10.1002/dad2.70380)
Supplement: Supplementary file 1 — Supporting Information: dad270380‐sup‐0001‐SupMat.docx [file DAD2-18-e70380-s001.docx]

Supplementary Materials

ereference

e1. Claessen T, Visser FCW, van Munster B, van der Flier WM, Jiménez‐Mausbach M, van Harten AC, et al. General practitioners’ perspectives on blood biomarkers for Alzheimer’s disease. Alzheimers Dement Diagn Assess Dis Monit. 2025;17(4):e70186.

Supplementary Table 1. Study-Level Metadata of Included Studies (n = 26), Including Design, Context, and Participant Characteristics

| **Author (Year, Country)** | **n** | **Participant Type** | **Design** | **Context** | **Age** | **% Female** | **Ethnicity** | **Education** |
| --- | --- | --- | --- | --- | --- | --- | --- | --- |
| **Aspö (2024, Sweden)** | 15 | Patients | Qual | Routine clinical care | Mean 60.8 yrs (range 50–72) | 53% | NR | Mean 14.6 yrs (range 11–19) |
| **Bélanger (2022, USA)** | 196 | Care partners | Mixed | Research setting | <65yrs (15%), 65–74yrs (47%), 75–84yrs (34%) | 68% | 95% White | HS≤15%, Some college 28%, College+ 57% |
| **Bolsewig (2024, NL)** | 114 | Caregivers | Mixed | Hypothetical Context | Mean 64.3yrs (SD 11.4) | 69% | NR | NR |
| **Couch (2022, USA)** | 62 | Care partners | Mixed | Research setting | <65yrs (21%), 65–74yrs (52%), ≥75yrs (27%) | 76% | 55% White | NR |
| **Gadbois (2022, USA)** | 400 | Patients & caregivers | Mixed | Research setting | 65–74yrs (52%), ≥75yrs (47%) | 38% patients | 95% White | HS≤17%, College+ 58% |
| **Grill (2017, USA)** | 52 | Patients & caregivers | Qual | Routine clinical care | Mean 73.1yrs (SD 10.3) | 58% | 87% White | Mean 17.2 yrs (SD 3.7) |
| **Hazan (2023, UK)** | 38 | Patients & HCPs | Mixed | Routine clinical care | Mean 74yrs (SD 8.5) | 65% patients | NR | NR |
| **James (2020, USA)** | 404 | Patients & caregivers | Mixed | Research setting | Mean 74.6yrs (SD 5.5–5.8) | NR | 96% White | HS≤14%, College+ 58% |
| **Kim (2023, USA)** | 68 | Patients & caregivers | Qual | Research setting | Mean 73.4yrs (SD 8.0) | 44% | 85% White | 59% graduate degree |
| **Kunneman (2017a, NL)** | 13 | HCPs | Qual | Routine clinical care | NR | 54% | NR | NR |
| **Kunneman (2017b, NL)** | 22 | Patients & caregivers | Qual | Routine clinical care | Patients mean 69yrs (SD 6.6) | 27% patients | NR | Low/middle/high (not quantified) |
| **Linden (2024, NL)** | 53 | Patients & HCPs | Qual | Routine clinical care | Patients mean 72yrs (63–82) | 40% patients | NR | Low 33%, Middle 33%, High 33% |
| **Lingler (2018, USA)** | 59 | Patients & caregivers | Qual | Research setting | Mean 72.9yrs (SD 8.9) | 63% | 87% White | 80% > high school |
| **Lohmeyer (2021, Germany)** | 44 | Patients &caregivers | Qual | Routine clinical care | 51–70yrs (n=5), >70yrs (n=7) | 42% patients | NR | Secondary–university levels |
| **O'Brien (2024, USA)** | 30 | HCPs | Qual | Routine clinical care | <40yrs (33%), 40–49yrs (17%), ≥50yrs (50%) | 53% | 77% White | NR |
| **Patel (2024, Canada)** | 65 | Patients & caregivers | Qual | Routine clinical care | Median 63yrs (IQR 56–68) | 59% patients | 86% White | 82% post-secondary |
| **Smith (2024, USA)** | 88 | Patients | Qual | Routine clinical care | Mean 71.6yrs (SD 7.9) | 45% | 92% White | 74% bachelor’s degree or higher |
| **Suridjan (2023, USA, China, UK, Germany, Spain, France)** | 215 | HCPs & payers, advisory board members | Qual | Implementation Context | 3–35 yrs experience | NR | NR | NR |
| **Swallow (2020, UK)** | 26 | HCPs | Qual | Routine clinical care | NR | NR | NR | NR |
| **Tromp (2021, NL)** | 15 | HCPs | Qual | Routine clinical care | 8–35 yrs experience | 40% | NR | NR |
| **van Gils (2022, NL)** | 17 | Patients &caregivers | Mixed | Routine clinical care | Mean 68yrs (SD 6) | 57% | NR | NR |
| **van Maurik (2019, NL)** | 36 | Mixed stakeholders | Mixed | Routine clinical care | NR | NR | NR | NR |
| **Vanderschaeghe (2017, Belgium)** | 38 | Patients | Qual | Research setting | Mean 70.9yrs (SD 6.6) | 42% | NR | Mean 13.2 yrs (SD 3.7) |
| **Vanderschaeghe (2019, Belgium)** | 40 | Mixed stakeholders | Qual | Research setting | 40–75 yrs (varied groups) | 0%-83% | NR | Secondary-master’s levels |
| **Visser (2020a, NL)** | 100 | Patients & HCPs | Mixed | Routine clinical care | Mean 70yrs (SD 11) | 41% patients | NR | NR |
| **Visser (2020b, NL)** | 23 | Patients & HCPs | Qual | Routine clinical care | Mean 73yrs (SD 9) | 46% patients | NR | NR |

HCPs, healthcare professionals; HS, high school; Mixed, mixed-methods; NL The Netherlands; NR, not reported; Qual, qualitative; SD, standard deviations; UK, United Kingdom; USA, The United States of America

Supplementary Table 2 The Critical Appraisals Skills Programme (CASP) qualitative checklist domains with explanations of assessment

| Domain | Explanation of Assessment |
| --- | --- |
| Clear Statement | **Was there a clear statement of the aims of the research?** We assessed whether each included paper had clearly reported their research objective and whether this was adequately framed amongst the research landscape. |
| Qualitative Appropriate | **Is a qualitative methodology appropriate?** We assessed whether a qualitative methodology *in general* (e.g. focus groups) was suitable to investigate the phenomena of interest (here, primarily subjective phenomena such as feelings and justifications of behaviours). |
| Research Design | **Was the research design appropriate to address the aims of the research?** We assessed whether the *specific* chosen methodology (e.g. questionnaires of patients) was appropriate to investigate the phenomena of interest (e.g. conflict with their caregivers). |
| Sampling | **Was the recruitment strategy appropriate to the aims of the research?** We examined how participants had been recruited into each study and whether this was appropriate to the research aims (e.g. recruitment of clinicians at conferences vs. through the network of memory clinics local to the research institution). |
| Data Collection | **Was the data collected in a way that addressed the research issue?** We examined multiple factors for this domain, including the justification of data collection technique, consideration of data saturation, and whether changes had been made to the data collection regime mid-way through the study. |
| Reflexivity | **Has the relationship between researcher and participants been adequately considered?** We examined whether workers had explicitly addressed their own roles and biases during the conduct of the study. |
| Ethics | **Have ethical issues been taken into consideration?** We reviewed whether each study had sought ethical approval, or had obtained explicit informed consent. |
| Data Analysis | **Was the data analysis sufficiently rigorous?** We scored papers on the basis of our confidence in their data analysis process. Papers scoring positive for this domain had chosen and explained an appropriate technique for qualitative data analysis (e.g. thematic analysis) and had drawn conclusions that were adequately supported by the primary data and accounted where indicated for outlying or contradictory data. |
| Discussion of Findings | **Is there a clear statement of findings?** Papers were examined with regard to the discussion of the conclusions of their analysis and the framing of these findings in the context of the research question. We reviewed whether papers had considered the strengths and limitations of their findings. |
| Value | **How valuable is the research?** We considered whether the work and findings of each paper, taken broadly, added meaningfully to the extant research literature. |


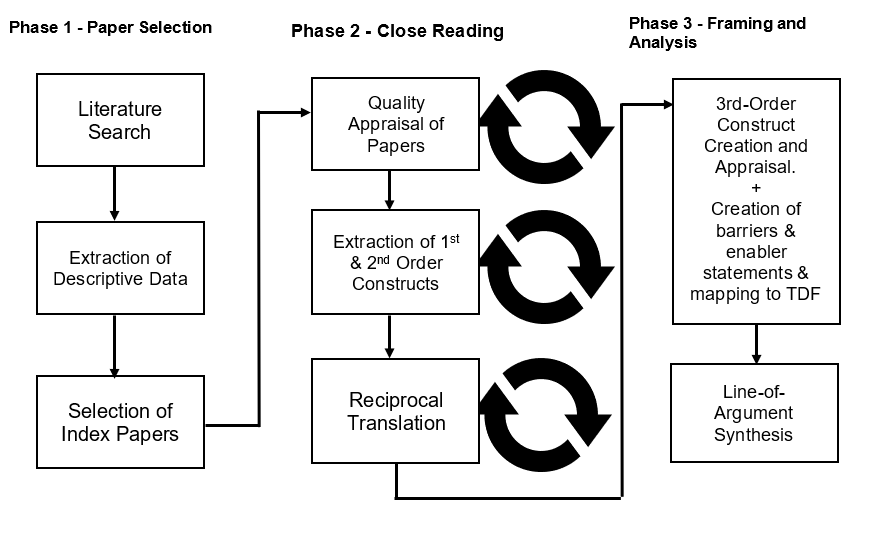


**Supplementary Figure 1.** Diagram of methods. Circular arrows are used to demonstrate the iterative nature of data analysis and synthesis steps.

1.

dementia/ or alzheimer disease/

2.

(Cogniti* adj2 (impair* or decline* or loss* or disorder* or deteriorat* or dysfunction*)).tw,kf.

3.

dementi*.tw,kf.

4.

(alzheimer* or alzeimer* or (cortical adj4 sclerosis)).tw,kf.

5.

1 or 2 or 3 or 4

6.

((biomarker* or marker*) adj3 (blood or plasma or early diagnos?s)).tw,kf.

7.

((cerebro spinal or cerebrospinal) adj2 fluid*).tw,kf.

8.

(early detection adj6 alzheimer*).tw,kf.

9.

("diagnosis and management" adj3 dementia).tw,kf.

10.

6 or 7 or 8 or 9

11.

Cerebrospinal Fluid/

12.

exp Biomarkers/

13.

exp Blood/

14.

exp Plasma/

15.

early diagnosis/

16.

13 or 14 or 15

17.

12 and 16

18.

(amyloid PET or scan or scans or amyloid imaging or amyloid tracer* or positron emission tomography or FDG-PET or fluorodeoxyglucose-positron emission tomography-CT or perfusion SPECT or single-photon emission CT or diagnostic test* or p-tau181 or AD biomarker*).tw,kf.

19.

10 or 11 or 17 or 18

20.

5 and 19

21.

exp qualitative research/

22.

interview/

23.

exp "Surveys and Questionnaires"/

24.

(interview* or survey* or qualitative or questionnaire* or focus group*).tw,kf.

25.

21 or 22 or 23 or 24

26.

exp Decision Making, Shared/

27.

((physician* or clinician* or doctor* or carer* or caregiver* or care giver* or family or patient* or individual or individuals) adj4 (perception* or preference* or view or views or opinion* or decision making or shared decision* or experience* or reaction* or knowledge or attitude* or diagnostic disclosure)).tw,kf.

28.

27 or 25

29.

20 and 28

Supplementary MEDLINE Search Strategy and Conceptual Framework

The search strategy combined four key concept domains:

1. Population: Dementia, Alzheimer’s disease, and cognitive impairment

2. Biomarker Investigations: Blood-based biomarkers, Cerebrospinal fluid, amyloid PET, imaging

3. Study Design: Qualitative research, interviews, surveys, focus groups, and questionnaires

4. Stakeholder Perspectives: Perceptions, attitudes, experiences, decision making, shared decision making, and disclosure

Subject headings (e.g. MeSH) were combined with free-text keywords using Boolean operators (AND/OR). Adjacency operators (e.g. adj2) were used to incorporate relevant phrase variations

Supplementary Table 3: Translation of First-, Second-, and Third-Order Constructs in the Meta-Ethnography

| Third-order construct | Second-order construct | First-order construct |
| --- | --- | --- |
| **CONCEPT 1 - EXPECTATIONS AND MOTIVATIONS OF STAKEHOLDERS** | | |
| **Caregivers and patients place different value on the need for biomarker testing.** | the possibility to take action in time (25) | "So I can take measures into my own hands and make the right decisions on time.” Caregiver (25) |
|  | Information for self(27) | “I was glad that he was doing something about it to find answers. And sometimes he’s reluctant to participate or go to an appointment, but I remind him, I said ‘by not going doesn’t change anything, it gives information and you and I will both be better prepared for what’s going to happen’” Caregiver (27) |
|  | strong encouragements or even pressure from family members(32) | "It was my daughter who signed me up [to go to the doctor], it’s as simple as that and so I did it" Patient (32) |
|  | Initiating the Process of Seeking a Scan or Diagnosis(27) | “My husband was the one who said, ‘I think you should ask the doctor if they could see if there’s anything wrong here,’ and so I did.” Patient(27) |
|  | Tension between family caregivers and tested persons(34) | The “hated daughter” Caregiver(34) |
| **If caregivers and patients decide to seek biomarker testing, their motivations may differ.** | Information for self (27) | “We just thought that, you know, there’s the opportunity to have some direction or understanding of what’s happening, why wouldn’t we purchase it?” Caregiver(27) |
|  | help receive the necessary care (32) | “then I said to my husband, why not have it checked out, then we will know for sure. And if you do have it, at least you can start taking medication quickly.” Caregiver (32) |
|  | Motivated by desire to plan ahead and/or make informed decisions(9) | “I understand what the test does, what you’re looking for and, uhm, it’s going to help me determine what I’m going to do with my life in the future.” Patient (9) |
| **Satisfaction with testing depends on how well participants’ expectations matched with the reality of what testing can provide.** | expressed relief (28) | “Well, it was, me personally, it was what I expected. I didn’t really think it was anything other than Alzheimer’s.” Patient (28) |
|  | The expectations and motivations of individuals for visiting the memory clinic strongly impacted their experience. (23) | "Part of it is finding out what it's about, what's wrong, so you can do something about it, and kind of find your way back, or get it back, that memory and that cognition that you've had in the past as well. [...] But those are kind of high expectations [...]." Patient(23) |
|  | hoped the scan would remove the “constant uncertainty” associated with not having a diagnosis(27) | “The doctor had discussed with us whether we thought it would be helpful if there were a medical procedure or test that would say definitely whether he had Alzheimer’s disease or did not, and we both thought that that would be an excellent idea to have this knowledge….I think that the definitive diagnosis, it’s nothing but helpful.” Caregiver (27) |
|  | the diagnostic work‐up impacted people psychosocially (23) | Was this it, well, what happens now? What, what should I do next, what should I do with this memory problem? How, yes, what should I do? Who is it that should answer about what is to come, or about the future, or what opportunities there are to either continue to look for causes or to do something about it. [...] But, but now what? What should I do now? Patient (23) |
| **Prior experiences and personal context shape the desire for biomarker testing.** | Reasons for Wanting the Scan or Diagnosis - Cognitive symptoms, family history(27) | “She initiated the contact with the doctor because she was concerned about her memory. Partially, and probably the major portion of it was because of her experience with her sister’s Alzheimer’s.” Caregiver (27) |
| **CONCEPT 2 - ATTACHED MEANING OF THE TEST RESULT** | | |
| **Willingness to undergo testing is shaped by perceptions of perceived availability or unavailability of treatments or interventions.** | gratitude for the confirmation and ability to plan ahead (24) | “I’m certainly grateful that we’re on that ... I’m not grateful we have the diagnosis but that the diagnosis was found sooner than later and that they can continue to treat him and do what we can with some ongoing ... at this.” Caregiver(24) |
|  | benefits of emerging pharmacologic treatments (7) | “the diagnosis is much more important now that we have potential therapies in the pipeline.” HCP(7) |
|  | Anticipatory guidance and care planning (7) | "Until there are some therapeutic options that get informed by the testing, I’m not sure it’s overly beneficial.” HCP (7) |
|  | treatment unavailability(25) | "I would not like to know already now that I am developing young-onset dementia [. . . ], because there is no treatment yet.” Patient (25) |
|  | start with medications or “cures” as soon as possible(32) | We decided with the company doctor at work (.) to have it checked out and possibly cured. Patient (32) |
|  | Hope for therapy(34) | “For, uh, it is, I think, in medical circles it is absolutely undisputed that the earlier you start a treatment, especially a drug treatment, the more the process slows down.” Caregiver (34) |
|  | morally acceptable (34) | “To my mind, such a test is all very well. But when there are no possibilities for healing, one can say: ‘You can still stop the process’. Then I don’t know if you would tell people without further ado. I’ve got my doubts there.” Caregiver (34) |
| **Psychosocial impacts vary between groups and from relief to consideration of suicide.** | Consequences of an abnormal test result (25) | “The danger is that if you get sick, getting sick is becomes your own fault.” Caregiver (25) |
|  | Negative outlook (36) | “They confirmed I have ALZ so my brain will continue to shut down affecting my abilities and my body’s functions until ultimately I no longer breath[e]. There is currently no cure and minimal helps.” Patient (36)  “scary, devastating” Patient (36) |
|  | factors that influenced the decision to initiate diagnostic testing (32) | “Because he was actually fired on the spot, you need to apply for unemployment benefits, but I wanted it to be sickness benefits, because I thought: a new job is out of the question. it has financial consequences.” Caregiver (32) |
|  | opportunity to motivate individuals to adopt a healthier lifestyle (23) | “Speaking of health effects, thinking of what one eats and drinks and that kind of stuff, that is affected of course, and I think more often about that now [...] my thoughts go back to those discussions I had with you [the clinic staff]. And now it's time to actually do it too.” Patient (23) |
|  | encouraged to improve their lifestyle (23) | "I have started exercising a bit, something I have never done before” Patient (23) |
|  | Positive outlook (36) | “I am hopeful. I have strong family support and I am enrolled in a clinical trial.” Patient (36) |
|  | planning of suicide (34) | “And I think that if you indeed have the probability... ... I think you can think more clearly about it with a certain distance. To organize things - If there is a pill that can end one’s life immediately or some [other] things, I think. But at least I have the option to think about it.” Caregiver (34) |
|  | potentially risky reaction to  a positive scan (9) | “I’m afraid in a way that he will maybe even try moving out or something because he keeps saying that he doesn’t want to be a burden.” Caregiver (9) |
|  | perceived harm (7) | “Alzheimer’s is such a charged topic, you could have people who overreact to the news” HCP (7) |
|  | other immediate actions (30) | “Now, what do I say to my kids, or what should they know?” Patient (30) |
|  | difficult emotions when the scan results did not align with their expectations (24) | “shocking,” “distressing,” and “devastating,” Caregiver (24) |
|  | definitive diagnosis (28) | “It was kind of expected I think. I was hopeful that it wouldn’t be the case, but I remember our family thinking ‘at least now we know’ and that was helpful. It identified it for us, I think.” Caregiver (28) |
|  | Relief was a recurring emotion (24) | “It relieved us, because we were really ... Because of the memory loss, we were really afraid that he did have Alzheimer’s, so we were very relieved.” Caregiver (24) |
|  | develop coping strategies (26) | “When that hits me and I’m just kind of freaking out, I go and open the picture of the PET scan on my desktop and say, ‘Oh yeah,’ and it helps me remind myself of what we’re dealing with. It's not him. It's the Alzheimer’s. I have this mantra, ‘It's not him. It’s the Alzheimer’s,’ and the PET scan helps me remember that. So I guess that's kind of, for me, it’s just a reminder that this is a real thing, not just he’s not just being weird.” Caregiver (26) |
| **All stakeholders perceive the possibility of stigma attached to a test result.** | Stigmatising (43) | “so from the moment onward that we disclosed, X got immediately labelled, and that is a really a stigma" Caregiver (43) |
|  | frustrated by memory problems which could not be attributed to dementia (26) | “At times, it’s very frustrating because of the memory loss. I keep hearing, he doesn't have Alzheimer’s. He doesn’t have dementia. It’s ADHD. That’s something not as serious. It’s something that we just have to deal with day-by-day.” Caregiver (26) |
|  | fallout from a false positive result (7) | “you don’t want to label someone with Alzheimer’s when they don’t have it.” HCP (7) |
|  | experiences of stigmatization by family members (34) | “Don’t worry! It’s only your dementia.’ And that was it then.” Patient (34) |
|  | difficulties coping with that label (32) | “But I found that really upsetting, I found it a quite upsetting label.” Caregiver (32) |
| **The diagnostic and prognostic value placed on the test result by patients and caregivers does not always align with the information that the test provides.** | possible treatments or lifestyle changes to stabilize or undo the patient’s cognitive decline (32) | “You do expect a bit more, perhaps that he could tell you that straight away (.) he is the doctor.” Caregiver (32) |
|  | natural progression of the disease (30) | “How does Alzheimer’s progress? I mean, like, today I feel okay, once in a while, I forget little things.” Patient (30) |
|  | need for more information on the prognosis of the disease (32) | “how will it develop, can you explain a bit more, well that will all be discussed at one of the later occasions I guess.” Patient (32) |
| **There is no consensus amongst HCPs on where AD is defined along a clinical to biological continuum.** | normal ageing (39) | "...Yes, I think that indeed makes my opinion different from someone who sees it as a disease [...] yes I mean Alzheimer’s is a part predisposition, a part environment, part aging, there are a lot of things.” GP (39) |
|  | clear distinction between the usefulness of these terms for clinical practice and in research(39) | “Dementia is . . . Alzheimer’s Disease is. . . . dementia is in any case a clinical condition, ‘so you’ll have to have symptoms for that. Alzheimer’s is of course a disease, so in that sense you could diagnose Alzheimer’s before someone has a dementia and notices that he is going to get it. I don’t see those people, luckily I would almost say, because it seems like a huge drama to tell people two years before they get dementia that they have Alzheimer’s Disease.” HCP (39) |
|  | Certainty of diagnosis (10) | “I wouldn't think it was a bad thing for everyone. The more information we have the between and for some patients it will change the diagnosis which will be helpful as well as making it more certain” HCP (10) |
| **CONCEPT 3 – SHARED DECISION MAKING AND RESULT COMMUNICATION** | | |
| **The decision to offer biomarker testing is typically made by HCPs, with shared input from patients and caregivers being more aspirational than commonly practiced.** | person-characteristics… influencing their decision (39) | “If you see someone aged 85 and you are going to do that, well, what do you want to achieve with that?" Neurologist (39) |
|  | Patient Characteristics(7) | “if somebody is over 75, there’s really little gain by making this diagnosis,” HCP (7) |
|  | Impact on Patient and Family(7) | “you don’t want to burden a patient before it’s necessary with that horrible...with the diagnosis that may indeed carry a difficult prognosis.” HCP (7) |
|  | steering patients in a certain direction (33) | “I definitely apply SDM. I think you must get someone motivated to undergo further diagnostic testing. If I refer someone who doesn’t want to, they’re not going to cooperate with that either, are they?” HCP(33) |
|  | raising the topic of testing (32) | “I believe that we didn’t even have an initial appointment because the GP said that tests would need to be done” Caregiver (32) |
|  | clinicians implicitly discouraged further biomarker testing by using diminutives (45) | “We can play all kinds of tricks” Patient(45) |
|  | Regarding the decision(s) about which tests to use, patients and caregivers felt these were mostly made by the clinician and without their own involvement. (32) | “no, you don’t have a choice, you don’t have a choice. You just have to take it all as it” Patient (32) |
|  | need for individualised communication in the decision-making process (33) | “It was nice to have a listening ear’” Patient(33) |
| **Pre-test counselling involves limited focus on the potential negatives of testing and patients may instead appraise the decision to test in the context of prior experiences of medical care.** | Easy process (9) | “Well I really didn’t think about it twice. I figured the more understanding there is, the better.” Patient (9) |
|  | comforting that clinicians made these decisions (32) | “no doubt they will do what is useful, so I just went into it blind and full of trust.” Patient(32) |
|  | Information for self (27) | “The doctor thought that my husband would be a very good candidate because at that point the neurologist didn’t know whether or not he had Alzheimer’s. My husband was very willing to do it.” Caregiver (27) |
|  | They trusted their GPs’ expertise and decisions or were not aware of the options available to them.(33) | “I trusted the information provided by the practice nurse was everything I needed to know” Patient(33) |
|  | drawbacks of amyloid PET were mentioned less often than potential benefits (9) | “I don’t see any reason not to do it” Patient (9) |
|  | Decision similar to and different from other medical decisions (9) | “I think it was pretty similar [to other medical tests] because both equate to understanding and knowing a little more than if I didn’t do it.” Patient (9) |
|  | contrasted this decision… with prior medical decisions (9) | "this is a choice. This was, for me, a lot more difficult than whether or not I should have heart surgeries" Caregiver (9) |
| **Adequate test result communication is a vital but challenging component of testing.** | strong need for information on the disease stage(32) | “I find that a bit silly: “look at that,” and I look and see a head on a screen, but you have no frame of reference, you don’t know what you are really seeing.” Patient(32) |
|  | struggled with the idea of a dichotomous result (30) | “It’s on a continuum—it’s not a plus or minus test, it’s a continuum and scale of one to ten, we were two —something like that?” Patient (30) |
|  | Interpretation(7) | “[I]f this could be a dichotomous result...that would be perfect. If... there’s some sort of risk spectrum...clear guidance as to the implications of the results on the patient’s expected outcome, some clear way to communicate to the patient what it means.” HCP(7) |
|  | shared the scan images with them and felt that this was helpful(28) | “When we were able to look at what the doctor represented as a normal scan and then we looked at my wife’s, it was very apparent that there was a difference.” Caregiver(28) |
|  | help patients and family members to understand the result (10) | "Displaying the data and where patient sits on that in a more user‐friendly way". HCP (10) |
|  | to take the scan with them (30) | “Can we have the results this center of doctor? Or maybe a copy them that I can give it to him.” Patient (30) |
|  | limited time (32) | "A very brief explanation, it felt as if you were back in the corridor with your diagnosis within 10 minutes" Caregiver (32) |
|  | left in the dark (32) | “A relatively short conversation with a clear opinion, you have the early stages of Alzheimer’s. But very little about the results of the tests, how well or badly you did (.) When we got home, we both had the feeling that we were actually missing an awful lot of information.” Patient (32) |
|  | value having a consultation with a psychologist or nurse (32) | “ And I did have the feeling that we needed to get away quickly, so we may not have much time to talk about it. (.) After that, we had a conversation with the medical psychologist, which was good, because they have more time. (.) and I do understand that the neurologist is not there to take care of you, it’s actually quite a formal conversation.” Caregiver (32) |
|  | greater heterogeneity as to the understanding and conceptualizing of the test results (34) | Others said that MCI “might become a problem”, that MCI was “not necessarily age-related” or that it “meant an increased risk for Alzheimer’s disease”. Caregiver (34) |
|  | Challenging - Physician thought normal aging (27) | “oh, it’s nothing. It’s just that you’re retiring and it’s a change of life,” Caregiver (27) |
|  | confusion about diagnostic terminology (29) | “According to the doctor, it was going to show whether or not he has Alzheimer’s. And it came out positive that he does not”. Caregiver (29) |
|  | equated amyloid positivity with an AD diagnosis(9) | “I want to know the defining thing on Alzheimer’s.so far [that] is that PET scan and that’s testing amyloid, right?” Patient (9) |
|  | misinterpretation that a positive scan result meant that the participant was positive for AD (30) | “And does that necessarily mean absolutely Alzheimer’s?” Patient (30) |
|  | expected the scan to provide information about the level of plaque deposition and/or the severity of the disease (28) | “[I expected to learn] if there was plaque present on my mom’s brain and how much was there. If it was there, how advanced it was.” Caregiver (28) |
|  | Clarification of results(30) | “Your definition of ‘significant’ would be comparable to what?” Caregiver (30) |
|  | Guidelines (7) | “[S]eeing recommendations for when to use the test and how to talk about it with patients would be helpful.” HCP (7) |
| **CONCEPT 4 – DESIRE FOR DIAGNOSTIC CERTAINTY** | | |
| **Participants express a desire to undergo testing as a means of providing diagnostic certainty.** | desire to receive a definitive diagnosis (28) | “We wanted to know whether I had Alzheimer’s or not. That was pretty much what I was pulling for, that they could determine whether I did.” Patient (28) |
|  | plasma AD biomarkers might aid clinical decision- making (7) | diagnosing AD “feels like a bit of a diagnostic exclusion,” and so plasma biomarker tests might enhance diagnostic certainty. HCP (7) |
| **Greater diagnostic weighting is placed on biomarker tests when they are invasive whereas this is reduced if the result conflicts with the clinical impression or other test results.** | information gained through the procedure (28) | “Well I think the importance again is that there’s kind of the physicality to it that we can see and that the doctor can see and be able to tell us, you know, this looks like it could be signs that my mom has, you know, early onset Alzheimer’s" Caregiver (28) |
|  | some diagnostic tests… provided more certainty on the diagnosis than other tests (32) | “[that lumbar puncture was] good, because it gives you certainty that it really is Alzheimer’s.” Caregiver (32) |
| **CONCEPT 5 – SYSTEMS AND PATHWAYS TO TEST DELIVERY** | | |
| **There is variability in perceived accessibility of biomarker testing between stakeholder groups.** | number of patients seeking memory evaluations far exceeds the number of available specialists (7) | “lack of opportunity to diagnose memory disorders and a real lack of access for people,” Geriatrician, HCP (7) |
|  | enable access to a diagnosis for those unable to see a specialist (7) | There are those with limited access who “desperately need a diagnosis for planning and resources,” so a blood test that could be used in primary care “would be helpful.” HCP (7) |
|  | logistical challenges associated with finding appropriate specialists (7) | “And then asked the family doctor to please give us a referral to him, and we had to wait at least four months to get in to see him. So that was probably the most difficult part of finding someone to help.” Caregiver (7) |
|  | Unmet need (37) | Both advisory boards and the initial survey revealed “missing patients” (AD patients without a timely diagnosis). HCPs (37) |
| **Variability in GP referrals to memory services is influenced by the GP’s clinical skill and knowledge.** | challenge of convincing doctors that their symptoms were different from normal aging (27) | primary care physicians “need to be more aware” Caregiver (27) |
|  | Straightforward Process - Team effort (27) | “I don’t think it was difficult to get the diagnosis. It was difficult to get a good doctor. But once he got to Dr. [Name], a neurologist, after that, things got better because she was knowledgeable, educated, caring of her patients.” Caregiver (27) |
|  | Challenging - Difficulties finding providers (27) | “Well, it took some perseverance. We went to our primary care first, several times..." “I don’t think family physicians pick it up right away. I think they need to be more aware of things like this. That slowed us down a lot in the beginning.” Caregiver (27) |
|  | Difficulties accessing services (27) | “ The whole way, it was a fight… I must’ve called 15 different numbers...He [Scan Recipient] actually was put on the drugs I think too late.” Caregiver (27) |
|  | straightforward process (27) | “it was not difficult. I talked to my primary care physician about having, being aware of short term memory loss and he connected to the doctor very quickly.” (27) |
|  | lack of consistency in reasons for referral or requests for confirmatory tests (37) | Not provided; HCPs |
| **HCPs’ willingness to use a biomarker test is impacted by the test properties.** | They felt the high PPV would allow a greater confidence in the test results and improve clarity and certainty regarding AD diagnosis (37) | Not provided; HCPs |
|  | “…a rule-out test could save time, money, and resources” HCPs (37) | Not provided; HCPs |
|  | Risk and burden of test (39) | “And I think that this kind of diagnostic work-up [conversation at home with MMSE] is much more pleasant for that lady and for that family, than hoisting someone like that in a bus to the hospital and pulling them through a scan and doing an amyloid measurement and an LP that does not add much more” Geriatrician, HCP (39) |
| **HCPs use biomarker tests in idiosyncratic ways.** | variable uptake of these tests in primary care (7) | “...like every other test, it would be used extensively by some and used not at all by others... we all know doctors who never met a test they didn't want to order, and we all know some physicians that are hesitant to order testing.” HCP (7) |
|  | Official guideline (39) | “I am not entirely up-to-date with the latest state of science in this area. So I follow the guideline, which is now just three years or a little older.” HCP (39) |
|  | Use in Primary Care (7) | “the only thing that’s going to bring some consistency to that would be clear cut studies and guidelines.” HCP (7) |
|  | limited experience evaluating patients for cognitive complaints (7) | “I’m not confident primary care physicians would use the test the way the test is designed” HCP (7) |
|  | Evidence (7) | “I would feel empowered to [use the tests] if I had appropriate guidance and confirmation of clinical utility and evidence base from my trusted experts locally and nationally.” HCP (7) |

Supplementary Table 4. Mapping of Qualitative Findings to TDF Domains: Barriers and Enablers for Alzheimer’s Disease Biomarker Testing with Supporting Illustrative Quotes

^a^ Enablers are underlined.

^b^Relevant Stakeholders: C, Caregivers; P, Patients; HCPs, healthcare professionals

AD, Alzheimer’s disease; GP, general practitioners (alternatively known as a primary care physician); HCPs, healthcare professionals; MCI, mild cognitive impairment

| **CONCEPT 1 - EXPECTATIONS AND MOTIVATIONS OF STAKEHOLDERS** | | | | | |
| --- | --- | --- | --- | --- | --- |
| **Third-order Construct** | **Barrier Enabler/Mixed^a^** | **TDF Domain** | **Relevant Stakeholders^b^** | **First-order Construct** | **Papers** |
| **Caregivers and patients place different value on the need for biomarker testing.** | Caregivers seek out testing in cases where patients can lack the motivation to do so (M) | Intentions | P,C | "So I can take measures into my own hands and make the right decisions on time.” Caregiver (25) | Bolsewig |
|  |  |  |  | “I was glad that he was doing something about it to find answers. And sometimes he’s reluctant to participate or go to an appointment, but I remind him, I said ‘by not going doesn’t change anything, it gives information and you and I will both be better prepared for what’s going to happen’” Caregiver (27) | Gadbois |
|  | Caregivers encourage patients to seek testing which can lead to tension between stakeholders (E) | Social Influences, Emotion | P,C | "It was my daughter who signed me up [to go to the doctor], it’s as simple as that and so I did it" Patient (32) | Kunneman (‘Patients’ and caregivers’ …’) |
|  |  |  |  | “My husband was the one who said, ‘I think you should ask the doctor if they could see if there’s anything wrong here,’ and so I did.” Patient (27) | Gadbois |
|  |  |  |  | The “hated daughter” Caregiver (34) | Lohmeyer |
| **If caregivers and patients decide to seek biomarker testing, their motivations may differ.** | Patients seek testing to gain information for “knowledge’s sake” and caregivers use it to validate their experiences (E) | Goals | P,C | “Then there’s folks like me who want all the information so that I can better make an informed decision somewhere along the way.” Patient (9) | Lingler |
|  |  |  |  | “I prefer to make decisions with as much information as I can get and I’ve always been that way.” Patient (9) | Lingler |
|  |  |  |  | “I want to know. [Even though] You might not be able to do anything about it.” Patient (9) | Lingler |
|  |  |  |  | “Yeah, and with him [her husband], nobody wants to believe that he has dementia. ‘What? He still does everything, he rides his bike, he dresses. He is fit, it seems. Be glad that he still is ... Isn’t he?’ Nobody believes it.” (34) | Lohmeyer |
|  | Caregivers use testing to inform care and treatment planning (E) | Behavioural Regulation | C | “But from a standpoint of managing her care and figuring out how best to take care of her with her symptoms, I feel like the scan was really positive in that it let me know she probably couldn’t go home and live by herself again and that I would really need to take her care in a direction that none of us had anticipated or could have predicted.” Caregiver (28) | Grill |
|  | Caregivers and patients perceive testing will lead to both positive outcomes (such as treatment access, clarity of symptoms and a sense of control and negative outcomes (M) | Beliefs about consequences | P, C | “We just thought that, you know, there’s the opportunity to have some direction or understanding of what’s happening, why wouldn’t we purchase it?” Caregiver (27) | Gadbois |
|  |  |  |  | “then I said to my husband, why not have it checked out, then we will know for sure. And if you do have it, at least you can start taking medication quickly.” Caregiver (30) | Kim |
|  |  |  |  | “I understand what the test does, what you’re looking for and, uhm, it’s going to help me determine what I’m going to do with my life in the future.” Patient (9) | Lingler |
| **Satisfaction with testing depends on how well participants’expectations matched with the reality of what testing can provide.** | Patients and caregivers have expectations that symptoms are due to Alzheimer's disease (AD) or dementia (E) | Knowledge | P, C | “Well, it was, me personally, it was what I expected. I didn’t really think it was anything other than Alzheimer’s.” Patient (28) | Grill |
|  | Patients have high expectations for testing and can express a sense of frustration if these are not met (B) | Beliefs about consequence, Emotion | P, C | "Part of it is finding out what it's about, what's wrong, so you can do something about it, and kind of find your way back, or get it back, that memory and that cognition that you've had in the past as well. [...] But those are kind of high expectations [...]." Patient (23) | Aspö |
|  |  |  |  | “The doctor had discussed with us whether we thought it would be helpful if there were a medical procedure or test that would say definitely whether he had Alzheimer’s disease or did not, and we both thought that that would be an excellent idea to have this knowledge….I think that the definitive diagnosis, it’s nothing but helpful.” Caregiver (27) | Gadbois |
| **Prior experiences and personal context shape the desire for biomarker testing.** | Patients and caregivers seek out a doctor’s appointment based on their personal experiences of AD in the family (E) | Intentions | P, C | “She initiated the contact with the doctor because she was concerned about her memory. Partially, and probably the major portion of it was because of her experience with her sister’s Alzheimer’s.” Caregiver (27) | Gadbois |

| **CONCEPT 2 - ATTACHED MEANING OF THE TEST RESULT** | | | | | |
| --- | --- | --- | --- | --- | --- |
| Third-order Construct | Barrier Enabler/Mixed^a^ | TDF Domain | Relevant Stakeholders ^b^ | Quotes | Papers |
| **Willingness to undergo testing is shaped by perceptions of perceived availability or unavailability of treatments or interventions.** | Caregivers are optimistic testing will enable access to care and treatment options (E) | Optimism | C | “I’m certainly grateful that we’re on that ... I’m not grateful we have the diagnosis but that the diagnosis was found sooner than later and that they can continue to treat him and do what we can with some ongoing ... at this.” Caregiver (25) | Bélanger |
|  | Both patients and HCPs vary in the degree to which they value the need for testing and this is related to their optimism or pessimism regarding treatments (M) | Optimism | HCP, C/P | “the diagnosis is much more important now that we have potential therapies in the pipeline.” HCP (10) | O'Brien |
|  |  |  |  | "Until there are some therapeutic options that get informed by the testing, I’m not sure it’s overly beneficial.” HCP (10) | O'Brien |
|  |  |  |  | "I would not like to know already now that I am developing young-onset dementia [. . . ], because there is no treatment yet.” Caregiver (26) | Bolsewig |
|  | Patients who lack knowledge about the limitations of available treatments are more likely to see the need for testing (E) | Knowledge, Beliefs about consequences | P | We decided with the company doctor at work (.) to have it checked out and possibly cured. Patient (31) | Kim |
|  | Some stakeholders value early diagnosis as this is perceived as facilitating early treatment (E) | Beliefs about consequences | C | “For, uh, it is, I think, in medical circles it is absolutely undisputed that the earlier you start a treatment, especially a drug treatment, the more the process slows down.” Caregiver (36) | Lohmeyer |
|  | Stakeholders raise ethical concerns with testing in the absence of disease modifying treatments (B) | Social professional role and identity | C | “To my mind, such a test is all very well. But when there are no possibilities for healing, one can say: ‘You can still stop the process’. Then I don’t know if you would tell people without further ado. I’ve got my doubts there.” Caregiver (36) | Lohmeyer |
| **Psychosocial impacts vary between groups and from relief to consideration of suicide.** | Some patients and caregivers experience hope and acceptance following a positive test result, whereas others experience despair (M) | Emotion | P, C | “I am hopeful. I have strong family support and I am enrolled in a clinical trial.” Patient (38) | Smith |
|  |  |  |  | “I think they put to rest any doubt about it being Alzheimer’s and so in that sense... Certainly on a cognitive level it was a relief in a sense to know that yes, this is it and this is what it is. Although we really figured that that’s what it was, it was still there was kind of a finality to it and that’s a relief.” Caregiver (29) | Grill |
|  |  |  |  | “It relieved us, because we were really ... Because of the memory loss, we were really afraid that he did have Alzheimer’s, so we were very relieved.” Caregiver (25) | Bélanger |
|  |  |  |  | “scary, devastating” Patient (38) | Smith |
|  |  |  |  | “They confirmed I have ALZ so my brain will continue to shut down affecting my abilities and my body’s functions until ultimately I no longer breath[e]. There is currently no cure and minimal helps.” Patient (38) | Smith |
|  | Caregivers and HCPs express concern over the anticipated emotional burden of a test result for the patient while patients express the same for their family. (B) | Emotion | P, C, HCP | “I’m afraid in a way that he will maybe even try moving out or something because he keeps saying that he doesn’t want to be a burden.” Caregiver (35) | Lingler |
|  |  |  |  | “Alzheimer’s is such a charged topic, you could have people who overreact to the news” HCP (10) | O’Brien |
|  |  |  |  | “Now, what do I say to my kids, or what should they know?” Patient (31) | Kim |
|  | Optimistic appraisals of a positive result may spur adoption of health-positive behaviours [add ASPO ref] and coping strategies [Couch et al., 2022] , whereas pessimistic appraisals may lead to suicidal ideation as an act of self-determination (35,37) (M) | Optimism and Memory, Attention, and Decision Processes | P, C | "I have started exercising a bit, something I have never done before” Patient (24)  “Speaking of health effects, thinking of what one eats and drinks and that kind of stuff, that is affected of course, and I think more often about that now [...] my thoughts go back to those discussions I had with you [the clinic staff]. And now it's time to actually do it too.” Patient (24) | Aspö |
|  |  |  |  | “When that hits me and I’m just kind of freaking out, I go and open the picture of the PET scan on my desktop and say, ‘Oh yeah,’ and it helps me remind myself of what we’re dealing with. It's not him. It's the Alzheimer’s. I have this mantra, ‘It's not him. It’s the Alzheimer’s,’ and the PET scan helps me remember that. So I guess that's kind of, for me, it’s just a reminder that this is a real thing, not just he’s not just being weird.” Caregiver (27) | Couch |
|  |  |  |  | “And I think that if you indeed have the probability... ... I think you can think more clearly about it with a certain distance. To organize things - If there is a pill that can end one’s life immediately or some [other] things, I think. But at least I have the option to think about it.” Caregiver (36) | Lohmeyer |
| **All stakeholders perceive the possibility of stigma attached to a test result.** | All stakeholders are concerned with stigma associated with a test result (B) | Social Influences, Social professional role and identity | P, C, HCP | “so from the moment onward that we disclosed, X got immediately labelled, and that is a really a stigma" Caregiver (45) | Vanderschaeghe |
|  |  |  |  | “At times, it’s very frustrating because of the memory loss. I keep hearing, he doesn't have Alzheimer’s. He doesn’t have dementia. It’s ADHD. That’s something not as serious. It’s something that we just have to deal with day-by-day.” Caregiver (27) | Couch |
|  |  |  |  | “you don’t want to label someone with Alzheimer’s when they don’t have it.” HCP (10) | O’Brien |
|  |  |  |  | “Don’t worry! It’s only your dementia.’ And that was it then.” Patient (36) | Lohmeyer |
|  |  |  |  | “But I found that really upsetting, I found it a quite upsetting label.” Caregiver (31) | Kim |
| **The diagnostic and prognostic value placed on the test result by patients and caregivers does not always align with the information that the test provides.** | Lack of knowledge of risk/prognostication (B) | Knowledge | P,C | “You do expect a bit more, perhaps that he could tell you that straight away (.) he is the doctor.” Caregiver (31) | Kim |
|  |  |  |  | “How does Alzheimer’s progress? I mean, like, today I feel okay, once in a while, I forget little things.” Patient (31) | Kim |
|  |  |  |  | “how will it develop, can you explain a bit more, well that will all be discussed at one of the later occasions I guess.” Patient (31) | Kim |
| **There is no consensus amongst HCPs on where AD is defined along a clinical to biological continuum.** | Variability in knowledge of AD definition (B) | Knowledge | HCP | "...Yes, I think that indeed makes my opinion different from someone who sees it as a disease [...] yes I mean Alzheimer’s is a part predisposition, a part environment, part aging, there are a lot of things.” GP (41) | Tromp, |
|  | Some HCPs question the value of biomarker testing as they perceive AD as a purely clinical diagnosis while others express a biomarker test will enhance diagnostic accuracy (10) (M) | Beliefs about consequences | HCP | “Dementia is . . . Alzheimer’s Disease is. . . . dementia is in any case a clinical condition, ‘so you’ll have to have symptoms for that. Alzheimer’s is of course a disease, so in that sense you could diagnose Alzheimer’s before someone has a dementia and notices that he is going to get it. I don’t see those people, luckily I would almost say, because it seems like a huge drama to tell people two years before they get dementia that they have Alzheimer’s Disease.” HCP (41) | Tromp |
|  |  |  |  | “I wouldn't think it was a bad thing for everyone. The more information we have the between and for some patients it will change the diagnosis which will be helpful as well as making it more certain” HCP (11) | Hazan |

| **CONCEPT 3 – SHARED DECISION MAKING AND RESULT COMMUNICATION** | | | | | |
| --- | --- | --- | --- | --- | --- |
| Third-order Construct | Barrier/Enabler/Mixed^a^ | TDF Domain | Relevant Stakeholders ^b^ | Quotes | Papers |
| **The decision to offer biomarker testing is typically made by HCPs, with shared input from patients and caregivers being more aspirational than commonly practiced.** | Some HCPs question the value of biomarker testing in patients with advanced age (B) | Beliefs about consequences | HCP | “If you see someone aged 85 and you are going to do that, well, what do you want to achieve with that?" Neurologist (41) | Tromp |
|  |  |  |  | “if somebody is over 75, there’s really little gain by making this diagnosis,” HCP (10) | O'Brien |
|  | HCP is concerned about the potential negative impact of providing a diagnosis too early (B) |  | HCP | “you don’t want to burden a patient before it’s necessary with that horrible...with the diagnosis that may indeed carry a difficult prognosis.” HCP (10) | O'Brien |
|  | Shared decision-making (SDM) is important to all stakeholders, but HCPs may use a more directive approach, limiting true patient involvement. (32) (33) (M) | Social professional role and identity, Memory, attention and decision processes | P, C, HCP | “I definitely apply SDM. I think you must get someone motivated to undergo further diagnostic testing. If I refer someone who doesn’t want to, they’re not going to cooperate with that either, are they?” HCP (34) | Linden |
|  |  |  |  | “no, you don’t have a choice, you don’t have a choice. You just have to take it all as it” Patient (33) | Kunneman |
|  |  |  |  | “It was nice to have a listening ear’” Patient (34) | Linden |
|  | Some GP referrals to memory services include an implicit endorsement of biomarker testing while dementia specialist HCPs’ language can explicitly convey a dismissive attitude towards biomarker testing (45) (M) | Social influences, Memory, attention and decision processes | P,C, HCP | “I believe that we didn’t even have an initial appointment because the GP said that tests would need to be done” Caregiver (33) | Kunneman |
|  |  |  |  | In addition, clinicians implicitly discouraged “We can play all kinds of tricks, including a PET scan or examining the cerebrospinal fluid. Those can then help me confirm, for example, that you have Alzheimer’s disease” Patient (47) | Visser  (‘Clinicians’ communication…’) |
| **Pre-test counselling involves limited focus on the potential negatives of testing and patients may instead appraise the decision to test in the context of prior experiences of medical care.** | Patients find the decision to undergo testing straightforward | Memory attention decision processes | P | “Well I really didn’t think about it twice. I figured the more understanding there is, the better.” Patient (35) | Lingler |
|  | Patients and caregivers have trust in HCP’s decision to suggest testing | Social influences | P,C | “no doubt they will do what is useful, so I just went into it blind and full of trust.” Patient (33) | Kunneman |
|  |  |  |  | “The doctor thought that my husband would be a very good candidate because at that point the neurologist didn’t know whether or not he had Alzheimer’s. My husband was very willing to do it.” Caregiver (28) | Gadbois |
|  |  |  |  | “I trusted the information provided by the practice nurse was everything I needed to know” Patient (34) | Linden |
|  | Patients do not always perceive negative consequences to testing | Beliefs about consequences | P | “I don’t see any reason not to do it” Patient (35) | Lingler |
|  | Patients found the decision to test comparable to past healthcare decisions in part due to a lack of knowledge about test rationale and implications (30,32) (E) | Memory attention decision processes, Knowledge | P | “I think it was pretty similar [to other medical tests] because both equate to understanding and knowing a little more than if I didn’t do it.” Patient (35) | Lingler |
|  |  |  |  | “I want to know the defining thing on Alzheimer’[s.so](http://s.so) far [that] is that PET scan and that’s testing amyloid, right?” Patient (35) | Lingler |
|  |  |  |  | “And does that necessarily mean absolutely Alzheimer’s?” Patient (31) | Kim |
|  | Caregivers found the decision to test more complex than past healthcare decisions (34)(M) |  | C | “[I expected to learn] if there was plaque present on my mom’s brain and how much was there. If it was there, how advanced it was.” Caregiver (29) | Grill |
|  |  |  |  | "this is a choice. This was, for me, a lot more difficult than whether or not I should have heart surgeries" Caregiver (35) | Lingler |
| **Adequate test result communication is a vital but challenging component of testing.** | Stakeholders express a lack of knowledge in interpreting the result (B) | Knowledge | P, C, HCP | “I find that a bit silly: “look at that,” and I look and see a head on a screen, but you have no frame of reference, you don’t know what you are really seeing.” Patient (32) | Kunneman |
|  |  |  |  | “It’s on a continuum—it’s not a plus or minus test, it’s a continuum and scale of one to ten, we were two —something like that?” Patient (31) | Kim |
|  |  |  |  | “[I]f this could be a dichotomous result...that would be perfect. If... there’s some sort of risk spectrum...clear guidance as to the implications of the results on the patient’s expected outcome, some clear way to communicate to the patient what it means.” HCP (10) | O’Brien |
|  | A printout summary report or visual representation of the biomarker result is valuable for patients and caregivers (E) | Memory, attention and decision processes | P, C, HCP | “When we were able to look at what the doctor represented as a normal scan and then we looked at my wife’s, it was very apparent that there was a difference.” Caregiver (28) | Gadbois |
|  |  |  |  | "Displaying the data and where patient sits on that in a more user‐friendly way". HCP (11) | Hazan |
|  |  |  |  | “Can we have the results this center of doctor? Or maybe a copy them that I can give it to him.” Patient (31) | Kim |
|  | Caregivers value having time to discuss results with other members of the multidisciplinary team (32), although in practice they often lack of time to discuss the test result (M) | Environmental context and resources | C, P | "A very brief explanation, it felt as if you were back in the corridor with your diagnosis within 10 minutes" Caregiver (33) | Kunneman |
|  |  |  |  | “A relatively short conversation with a clear opinion, you have the early stages of Alzheimer’s. But very little about the results of the tests, how well or badly you did (.) When we got home, we both had the feeling that we were actually missing an awful lot of information.” Patient (33) | Kunneman |
|  |  |  |  | “ And I did have the feeling that we needed to get away quickly, so we may not have much time to talk about it. (.) After that, we had a conversation with the medical psychologist, which was good, because they have more time. (.) and I do understand that the neurologist is not there to take care of you, it’s actually quite a formal conversation.” Caregiver (33) | Kunneman |
|  | HCPs provide Inadequate education on the differences between normal ageing, MCI (27,34,35) and symptoms of dementia (27) (B) | Knowledge | P, C | Others said that MCI “might become a problem”, that MCI was “not necessarily age-related” or that it “meant an increased risk for Alzheimer’s disease”. Caregiver (36) | Lohmeyer |
|  |  |  |  | “MCI was partly described as “actually a totally normal state”, “everything still all right”, as a “minor disruption” or “if at all, then only a tiny little bit of dementia”. Others said that MCI “might become a problem”, that MCI was “not necessarily age-related” or that it “meant an increased risk for Alzheimer’s disease”. Patients and Caregivers (36) | Lohmeyer |
|  |  |  |  | “oh, it’s nothing. It’s just that you’re retiring and it’s a change of life,” Caregiver (28) | Gadbois |
|  | Patients and caregivers may report their test result inaccurately (B) | Knowledge | P,C | “According to the doctor, it was going to show whether or not he has Alzheimer’s. And it came out positive that he does not”. Caregiver (30) | James |
|  | HCPs can use confusing language(30) when communicating the test result and may benefit from training (9). (M) | Skills | P,C,HCP | “Your definition of ‘significant’ would be comparable to what?” Caregiver (31) | Kim |
|  |  |  |  | “[S]eeing recommendations for when to use the test and how to talk about it with patients would be helpful.” HCP (10) | O’Brien |

| **CONCEPT 4 – DESIRE FOR DIAGNOSTIC CERTAINTY** | | | | | |
| --- | --- | --- | --- | --- | --- |
| Third-order Construct | Barrier/Enabler/Mixed^a^ | TDF Domain | Relevant Stakeholders ^b^ | Quotes | Papers |
| **Participants express a desire to undergo testing as a means of providing diagnostic certainty.** | Stakeholders request biomarker testing to remove uncertainty(9,27), sometimes by reducing reliance on exclusion-based diagnosis(9) (E) | Beliefs about consequences | P, C, HCP | “We wanted to know whether I had Alzheimer’s or not. That was pretty much what I was pulling for, that they could determine whether I did.” Patient (26) | Bolsewig |
|  |  |  |  | “diagnosing AD “feels like a bit of a diagnostic exclusion,” and so plasma biomarker tests might enhance diagnostic certainty.” HCP (10) | O’Brien |
|  | Patients express dissatisfaction with testing when it does not provide diagnostic certainty (B) | Emotion | P | “You must keep an eye on it, but it is not Alzheimer, uh, no Alzheimer is confirmed. I was quite glad about it, you see? But, as I said, this uncertainty doesn’t really... yes, make you more satisfied.” Patient (36) | Lohmeyer |
| **Greater diagnostic weighting is placed on biomarker tests when they are invasive whereas this is reduced if the result conflicts with the clinical impression or other test results.** | Difficulties can arise when the test result conflicts with the clinical impression, leading some HCPs to prioritise the clinical and potentially reducing their reliance on test findings in decision-making. (10) (B) | Beliefs about consequence,  Memory, attention and decision making processes | HCP | “There was one patient that it didn't impact on the diagnosis as it didn't help, it came back low but she clinically had a progressive AD” HCP (11) | Hazan |
|  |  |  |  | ‘when clinical presentation conflicts  with biology for example, biomarker or scan result, I would mark it down  and go with the clinical presentation’. HCP (11) | Hazan |
|  | Visible biomarker evidence strengthens trust in diagnosis and supports decision-making. (E) | Beliefs about consequences | P,C | “Well I think the importance again is that there’s kind of the physicality to it that we can see and that the doctor can see and be able to tell us, you know, this looks like it could be signs that my mom has, you know, early onset Alzheimer’s" Caregiver (29) | Grill |
|  |  |  |  | “[that lumbar puncture was] good, because it gives you certainty that it really is Alzheimer’s.” Caregiver (33) | Kunneman |

| **THEME 5 – SYSTEMS AND PATHWAYS TO TEST DELIVERY** | | | | | |
| --- | --- | --- | --- | --- | --- |
| Third-Order Construct | Barriers and Enablers^a^ | TDF Domain | Relevant Stakeholders^b^ | Quotes | Papers |
| **There is variability in perceived accessibility of biomarker testing between stakeholder groups.** | There is limited accessibility of testing and of a specialist assessment (B) | Environmental context and resources | P,C,HCP | “lack of opportunity to diagnose memory disorders and a real lack of access for people,” Geriatrician, HCP (10) | O’Brien |
|  |  |  |  | There are those with limited access who “desperately need a diagnosis for planning and resources,” so a blood test that could be used in primary care “would be helpful.” HCP (10) | O’Brien |
|  |  |  |  | “And then asked the family doctor to please give us a referral to him, and we had to wait at least four months to get in to see him. So that was probably the most difficult part of finding someone to help.” Caregiver (10) | O’Brien |
|  |  |  |  | Both advisory boards and the initial survey revealed “missing patients” (AD patients without a timely diagnosis). HCPs (39) | Suridjan |
| **Variability in GP referrals to memory services is influenced by the GP’s clinical skill and knowledge.** | Lack of knowledge of the evidence base for the test (B) | Knowledge | HCP | primary care physicians “need to be more aware” Caregiver (28) | Gadbois |
|  | Lack of training in recognising disease symptoms assessing cognitive complaints in primary care (B) | Skills | HCP | “Well, it took some perseverance. We went to our primary care first, several times..." “I don’t think family physicians pick it up right away. I think they need to be more aware of things like this. That slowed us down a lot in the beginning.” Caregiver (28) | Gadbois |
|  | There is variable ease in accessing a referral to specialist services(27) (M) | Environmental context and resources | P,C,HCP | “ The whole way, it was a fight… I must’ve called 15 different numbers...He [Scan Recipient] actually was put on the drugs I think too late.” Caregiver (28) | Gadbois |
|  |  |  |  | “it was not difficult. I talked to my primary care physician about having, being aware of short term memory loss and he connected to the doctor very quickly.” (28) | Gadbois |
| **HCPs’ willingness to use a biomarker test is impacted by the test properties.** | Blood biomarker tests are less resource intensive in terms of time to perform the test and cost (38)(E) | Environmental context and resources | HCP | “Respondents in the follow-up survey considered a rule-out test could save time, money, and resources” HCPs (39) | Suridjan |
|  |  |  |  | There are those with limited access who “desperately need a diagnosis for planning and resources,” so a blood test that could be used in primary care “would be helpful.” HCP (10) | O’Brien |
|  | The high positive predictive value of the test provides greater confidence in results, enhancing clarity and certainty in diagnosing AD (E) | Belief about consequences | HCP | “They felt the high PPV would allow a greater confidence in the test results and improve clarity and certainty regarding AD diagnosis” HCPs (39) | Suridjan |
|  | More invasive biomarker tests are less acceptable to some HCPs (B) | Memory attention decision processes | HCP | “And I think that this kind of diagnostic work-up [conversation at home with MMSE] is much more pleasant for that lady and for that family, than hoisting someone like that in a bus to the hospital and pulling them through a scan and doing an amyloid measurement and an LP that does not add much more” Geriatrician, HCP (41) | Tromp |
| **HCPs use biomarker tests in idiosyncratic ways.** | HCPs differ in their approach to when to order biomarker testing in relation to other investigations (M) | Memory attention decision processes | HCP | “...like every other test, it would be used extensively by some and used not at all by others... we all know doctors who never met a test they didn't want to order, and we all know some physicians that are hesitant to order testing.” HCP (10) | O’Brien |
|  | HCPs are unaware of guidelines(40) for use and there is a need for clear guidelines on biomarker use(9) M) | Knowledge | HCP | “I am not entirely up-to-date with the latest state of science in this area. So I follow the guideline, which is now just three years or a little older.” HCP (41) | Tromp |
|  |  |  |  |  |  |
|  |  |  |  | “the only thing that’s going to bring some consistency to that would be clear cut studies and guidelines.” HCP (10) | O’Brien |
|  | Non-specialist HCPs may misuse the test | Beliefs about capabilities | HCP | “I’m not confident primary care physicians would use the test the way the test is designed” HCP (10) | O’Brien |
|  | HCPs are more likely to test if there is confirmation from peer or authority influences | Social Influences | HCP | “I would feel empowered to [use the tests] if I had appropriate guidance and confirmation of clinical utility and evidence base from my trusted experts locally and nationally.” HCP (10) | O’Brien |
